# Decoding spatiotemporal features of emotional body language in social interactions

**DOI:** 10.1038/s41598-022-19267-5

**Published:** 2022-09-05

**Authors:** Johannes Keck, Adam Zabicki, Julia Bachmann, Jörn Munzert, Britta Krüger

**Affiliations:** 1 Neuromotor Behavior Lab, Department of Psychology and Sport Science, Justus-Liebig-University, Kugelberg 62, 35394 Giessen, Germany; 2 Center for Mind, Brain and Behavior-CMBB, Universities Marburg and Giessen, Marburg, Germany

**Keywords:** Computational neuroscience, Machine learning, Psychology and behaviour

## Abstract

How are emotions perceived through human body language in social interactions? This study used point-light displays of human interactions portraying emotional scenes (1) to examine quantitative intrapersonal kinematic and postural body configurations, (2) to calculate interaction-specific parameters of these interactions, and (3) to analyze how far both contribute to the perception of an emotion category (i.e. anger, sadness, happiness or affection) as well as to the perception of emotional valence. By using ANOVA and classification trees, we investigated emotion-specific differences in the calculated parameters. We further applied representational similarity analyses to determine how perceptual ratings relate to intra- and interpersonal features of the observed scene. Results showed that within an interaction, intrapersonal kinematic cues corresponded to emotion category ratings, whereas postural cues reflected valence ratings. Perception of emotion category was also driven by interpersonal orientation, proxemics, the time spent in the personal space of the counterpart, and the motion–energy balance between interacting people. Furthermore, motion–energy balance and orientation relate to valence ratings. Thus, features of emotional body language are connected with the emotional content of an observed scene and people make use of the observed emotionally expressive body language and interpersonal coordination to infer emotional content of interactions.

## Introduction

In everyday life, the expression of emotions is an essential part of human social interaction^1–3^. It is linked inseparably to the ability to observe, recognize, and evaluate the emotions of our conspecifics^4–8^.

Affect expression occurs through combinations of verbal and nonverbal communication channels^9^. To judge other people’s emotional states reliably, information can be decoded through nonverbal communication channels such as facial expressions or body movements and posture^10–17^. Up to now, most research in the field has focused on facial expressions. It has shown not only that people can express at least six different emotional states through their faces—anger, happiness, sadness, fear, surprise, and disgust—but also that these expressions demonstrate a high level of intercultural stability^18^. In recent decades, however, the focus of research has also shifted towards bodies. It has been suggested that recognition performance for bodily expressions is very similar to that for faces, and evidence has been provided that movements of the body or its segments also contain significant aspects of nonverbal communication^1,2,9,11,13,19^. For example, Michalak et al. have shown that gait patterns associated with sadness are characterized by reduced velocity, arm swing, and vertical motion of the head^2^. More recently, Poyo Solanas et al. demonstrated that fear is expressed through configurations of limb angles^9^.

These studies indicate that postural and kinematic features vary depending on the emotional state, and that they influence the perception of emotion categories^2,3,9,20–24^. Postural and kinematic features can be summarized under the heading emotional body language (EBL). EBL is described as behaviour used to express emotions via the whole body coordinated in its movements across multiple joints and often accompanied by a meaningful action^11,25^. Thus, the use of space or the arrangement of body posture, gestures, and trunk and arm movements are tools through which the body can express an emotion^2,9,17,20,21,24^. In contrast to facial expressions, EBL is often more action-oriented, and it can be identified even when the face is not clearly visible^15,16,26^. It further enables the observer to recognize a situation and simultaneously acknowledge the action undertaken by an individual^11,26^.

Consequently, EBL carries important information about not only the emotional state but also interindividual signalling^15,16^. Therefore, in this context, emotions can preferably be described as a dynamic relational process occurring between the individual and the environment^10^. In this vein, it has been demonstrated that contextual social information provided by interacting persons enhances the recognition of the emotional content of a scene and increases the observer’s confidence in their perceptual judgement^7,10^. Important contextual cues in social interactions might be embodied synchronization or proxemic measures such as distance and orientation^27–29^. Taken together, the aforementioned studies suggest that interaction-specific parameters also contribute to the perception and identification of emotions. However, up to now, it remains largely unknown which features drive emotion perception in social interactions on the intra- and interpersonal level.

Here, we investigate for the first time both levels of body features in social interactions and their influence on the perception of emotions from body language. We provide a quantitative description and computational framework of movement features in social interactions using univariate and multivariate analysis. In detail, we investigated intrapersonal EBL by computing several kinematic and postural features and relating them to emotion perception. Moreover, we focused on interaction-specific characteristics that contribute to emotion perception. We used 48 point-light displays (PLDs) of human interactions portraying four emotions (happiness, affection, sadness, and anger). Participants observed these stimuli and were asked to categorize both the depicted emotional content and the valence of the perceived stimulus. We quantified different intra- as well as interpersonal movement features and analysed differences between emotional categories. To evaluate the relative importance of each calculated feature in the classification of emotional content, we trained different decision tree classifiers. Finally, we explored the correspondence of both the perceived emotional content and the perceived valence of a scene to the computational features on intra- and interpersonal levels via representational similarity analysis (RSA).

## Materials and methods

### Participants

A total of 31 participants (16 women) with a mean age 23.58 ± 3.54 years participated in the experiment. None reported any history of psychiatric or neurological disorders and they had no history or current use of psychoactive medication. All procedures were approved by the local ethics committee of the Department of Psychology and Sports Science of the Justus Liebig University Giessen and adhered to the declaration of Helsinki. All participants provided written informed consent prior to participating.

### Stimuli

Stimuli were selected from a larger motion-capture data set^17^. Eight pairs of non-professional actors were instructed to perform an interaction portraying one out of four emotional scenes depicting either happiness, affection, sadness, or anger. To ensure a congruent behavioural pattern, actors were given a script of emotional situations and directed specifically to perform the same emotion. They were instructed to express their emotions intuitively within the context of the given situation, thereby allowing freedom and enhancing the variability of expression^17^. Interactions were recorded with an optical motion capture system (Vicon Motion Systems, Oxford, England) operating at 100 Hz. MATLAB software (Mathworks, Natick, MA) was used to create video files of 4-s sequences from the original coordinate 3D (C3D). In each video, 15 markers per person were plotted as white spheres on a black background to present a standard PLD model^30^.

The final stimulus selection was based on prior validation of emotion category and perceived valence from 24 participants who did not take part in the present experiment. Valence was judged on an 11-point scale ranging from − 5 (*extremely negative*) to + 5 (*extremely positive*). There were two validation criteria: first, at least 50% of the participants had to recognize the displayed emotion (e.g., anger); second, the second-highest emotion rating should not exceed 25%. This allowed us to identify and exclude ambiguous scenes in which a specific emotion could not be recognized reliably. After validation, 12 stimuli that met both criteria were selected randomly for each emotion category. This resulted in a set of 48 (4 emotions × 12 scenes) stimuli. For more information on stimulus creation and validation, see Supplementary Figs. S1, S11 and ^17^.

### Experimental procedure

Prior to the present experiment, participants were given instructions and acquainted with the task. They subsequently performed a test run containing 12 trials that were not included in the main experiment.

**Figure 1:**
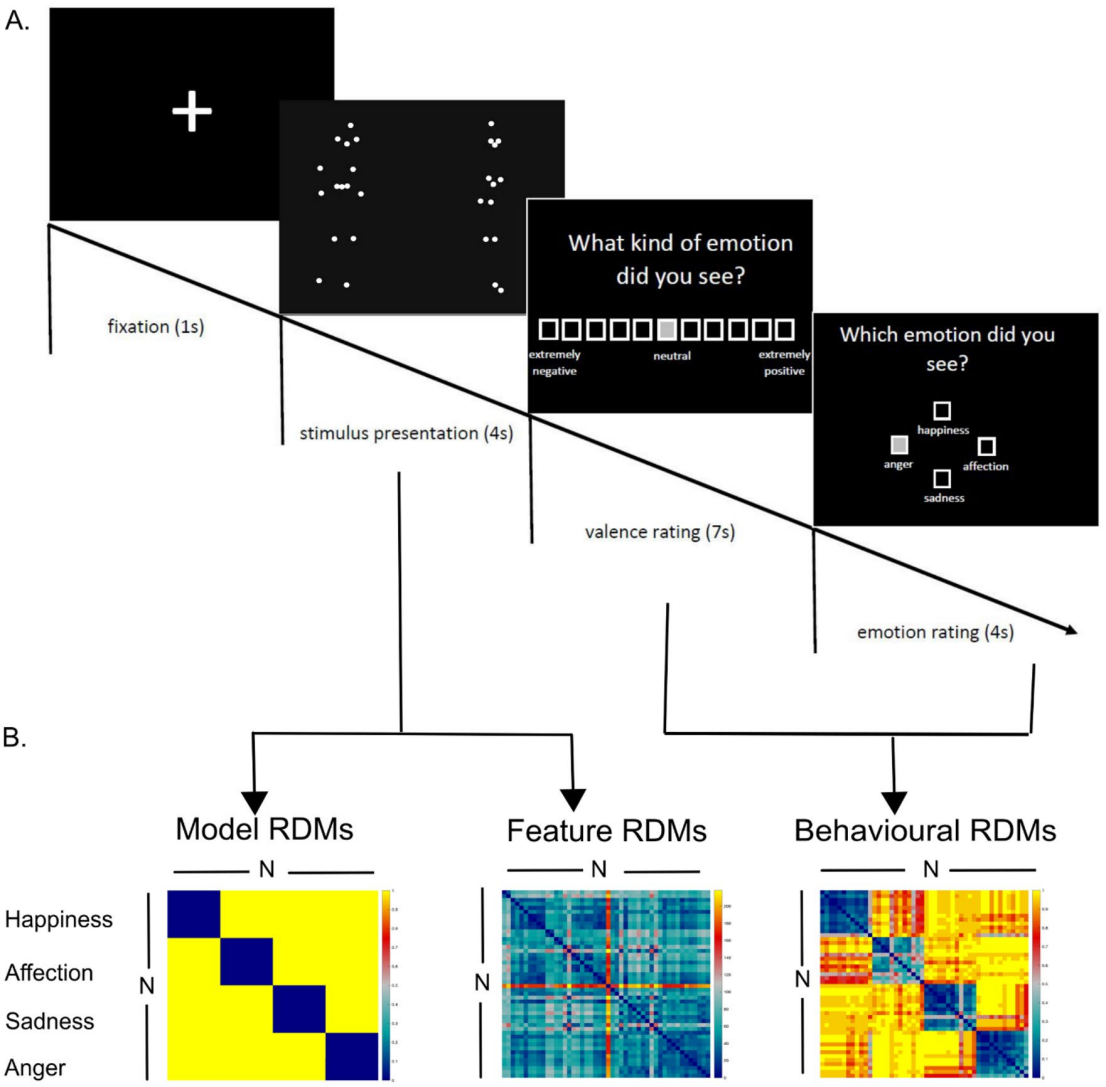
Experimental timeline and RDM creation. (**A**) Temporal structure of one trial. (**B**) Three different RDM types were created: first, model RDMs assuming categorical differences between emotions and valences by using binary variables (0 if identical, 1 otherwise); second, feature RDMs representing each parameter on the intra- and interpersonal level using Euclidean distance as similarity measure; third, behavioural RDMs using binary variables for emotion (1 if correct, 0 otherwise) and Euclidean distance for valence ratings.

In the experiment, each sequence was presented once, resulting in a series of 48 sequences. Sequences were displayed in a pseudo-randomized order on a 12-in. screen (refresh rate 60 Hz). The distance between each test person’s eyes and the screen was approximately 40 cm. Each trial started with a fixation phase (1 s) followed by a stimulus sequence (4 s) and two behavioural ratings. After observing this sequence, participants were asked to assess the emotional valence of the videos on the same scale that had been used for stimulus validation (7 s). The second step was to sort emotions into one of the following categories: happiness, affection, sadness, or anger (4 s) (Fig. 1A).

### Feature definition

To investigate EBL characteristics that drive the perceptual judgement on an intra- and interpersonal level, we calculated several features using MATLAB software. From the 15 markers displayed, we chose 13 anatomical points (excluding sternum and sacrum) that presented anatomical landmarks on the upper body (including shoulders, elbows, wrists, and head) and the lower body (including hips, knees, and ankles). Features were calculated from the x, y, and z coordinates.

On an intrapersonal level, the three kinematic features (calculated for each anatomical point) addressed *velocity, acceleration*, and *vertical movement*. We implemented *symmetry*, *limb angles* (shoulder, elbows, hips, knees), *limb contraction* (distance from head to wrist and ankles), *volume,* as well as its *standard deviation (volume STD)* as postural features^9,24^. Each feature was calculated within each of the 400 frames and averaged across time and actors.

In a next step, we computed 12 interpersonal parameters. Proximity measures included *interpersonal distance* (*IPD*) and its variance over time (*IPD STD*), the percentage of time spent in the personal space of the other agent *(personal space),* as well as *interpersonal orientation* (*IPO*) and the ratio of orientation from one person to another to detect imbalances *(IPO balance)* in which the persons are turned towards each other^9,28,31,32^. To investigate how the spatial distance between two people affects velocity, acceleration, limb angles, and limb contraction (with included time information), we correlated these measures with the distance profile (*distance correlations*). We also examined the synchronization of the velocity and acceleration profiles (*synchronization velocity & acceleration)*^29,33^. Finally, we calculated the proportion of the displayed motion energy *(motion–energy balance)* of each person^9,20,34^. For more detailed information on feature definitions and calculations, see Table 1, supplementary information, and^35^.

**Table 1:** Summary of interaction-specific intrapersonal and interpersonal features calculated with the SAMI toolbox^35^.

	Abbr	Short description
Intrapersonal Features		
Acceleration	ACC	Derived from the calculated velocity
Velocity	VEL	Marker from the 3-dimensional motion trajectories divided by the according time interval (1/100 Hz = 10 ms)
Vertical Movement	VM	Absolute amount of displacement of each marker along the z-axis of subsequent frames
Volume	VOL	Multiplication of the distances between the minimum and maximum anatomical point of a agent along the x-, y-, and z-axes for each frame,
Volume Standard Deviation	VOL STD	respectively the standard deviation
Symmetry	SM	Symmetry contains height difference in z direction, the distance as well as circular segment to a predefined line of symmetry for each agent from corresponding left and right anatomical points
Limb Angles	LA	Shoulder (shoulder to elbow and hip), elbow (elbow to shoulder and wrist), hip (hip to shoulder and knee), and knee joint angles (knee to hip and ankle).
Limb Contraction	LC	Distances from the left and right ankle and the left and right wrist to the head

Interpersonal Features		
Average of Interpersonal Distance	IPD	Spatial distance between both agents. Calculated as the mean over time
Variance in Interpersonal Distance	IPD STD	respectively the standard deviation.
Average of Interpersonal Orientation	IPO	Time spent facing each other + time spent by one agent facing the other and vice versa.
Balance of Interpersonal Orientation	IPO BAL	Absolute value of the difference between orientation times of each agent divided by the sum of orientation times; higher value indicating greater balance level
Correlation between Spatial Distance and Velocity	DC VEL	Relationship of spatial distance between both agents and mean velocity of whole-body movements
Correlation between Spatial Distance and Acceleration	DC ACC	Relationship of spatial distance between both agents and mean acceleration of whole-body movements
Correlation between Spatial Distance and Volume	DC VOL	Relationship of spatial distance between both agents and volume
Correlation between Spatial Distance and Limb Contraction	DC LC	Relationship of spatial distance between both agents and limb contraction
Synchronization of Velocity	SYNC VEL	Correlation between agent’s velocity profiles over time
Synchronization of Acceleration	SYNC ACC	Correlation between agent’s acceleration profiles over time
Motion Energy Balance	ME BAL	Difference between amount of body movement (sum of averaged inter-frame Euclidean displacements of each marker) from each agent divided by the total amount of body movement in the scene; higher value indicating greater balance level
Personal Space	PS	Time spent in the personal space (within one arm length) of the interactive partner

For a detailed explanation, see supplementary information.

### Data analysis and statistics

As a first step, we calculated the recognition rates (accuracy) of stimuli for each emotional category by comparing the target emotion with the behavioural response. To ensure a sufficient degree of stimuli recognizability, we tested each emotional category against chance (25%) using Bonferroni-corrected one-sample *t* tests.

### Influence of emotional categories

We tested for the emotion specificity of EBL features with a one-way ANOVA. The intrapersonal and interaction-specific features calculated from each stimulus were averaged across anatomical points and used as input. The ANOVA contained a four-level factor of emotion (happiness, affection, sadness, anger). Alpha was set at 0.05 for all statistical tests and post hoc pairwise comparisons were Bonferroni-corrected. Due to violations of the normal distribution in the values of interaction-specific features (*distance correlation, synchronization*), we normalized our data with a Fisher *Z* transformation^37,38^.

### Emotion classification with decision trees

To evaluate the relative importance of each calculated feature in the classification of emotional interactions, we trained decision tree classifiers using Matlab Statistics and Machine Learning Toolbox (Version 11.6). Classification of stimuli was based on the weighted majority of multiple decision trees (bootstrap-aggregating approach) to avoid overfitting and enhance generalization^24,39,40^.

Three different classifiers were trained for classification of emotions using averaged time information and averaged anatomical landmarks with different predictors: (1) M1 = intrapersonal features, (2) M2 = interpersonal features, and (3) M3 = combination of the two feature sets (M1 + M2).

To minimize the influence of randomly splitting the displayed 48 stimuli into the training and the validation dataset, we used leave-one-out-cross validation to estimate the performance of the different classifiers. To avoid imbalanced datasets and hence bias, each category was presented equally in training and test data (leave one stimulus out per category). For more information, see Supplementary Fig. S2.

### Representational similarity analysis

We used representational similarity analysis (RSA)^41,42^ to characterize the relationship between the perceptual ratings and computed EBL feature sets for each of the 48 stimuli. By relating the stimuli to each other and arranging the values horizontally and vertically in the same order, we created a symmetrical representational dissimilarity matrix (RDM) (48 × 48). Each entry describes the relation between two stimuli. In the main diagonal, the stimuli values are compared with themselves, resulting in a diagonal defined as zeros.

In a first step, we created two different model RDMs by assuming a categorical distinction between the emotion and the valence category of the stimuli. Therefore, the dissimilarities between identical categories were 0 and those between different categories were 1 (Fig. 1B).

Second, we calculated 31 individual single-subject RDMs for emotion categorization by also using binary variables (0 if identical emotional rating, 1 otherwise). Furthermore, we used individual valence ratings to create RDMs in which each cell corresponded to the pairwise absolute difference. Here and in the following step, we used the Euclidean distance measure (Fig. 1B)^24,35^.

To test which of the features related to the geometry of the model RDMs and the behavioural rating RDMs, we built feature RDMs representing the intrapersonal and interpersonal level (Fig. 1B). This step resulted in eight intrapersonal RDMs and 12 interpersonal RDMs.

To describe and test the relationship between all RDMs, we calculated a matrix of pairwise correlations (Kendall's τ_A_) between model and feature RDMs separately on the intrapersonal and interpersonal level. To account for multiple testing, we applied Bonferroni corrections based on the number of features in each set. We used multidimensional scaling (MDS) to gain a graphical impression of representational distances (computed as 1 − Kendall's τ_A_).

Furthermore, each feature RDM was tested against the behavioural RDMs using Kendall's τ_A_ for emotion categorization and Pearson correlation coefficients for valence ratings. Multiple testing was Holm–Bonferroni corrected, and the false-discovery rate was set at 0.05. The variance within the emotions and valence ratings across participants was represented by the noise ceiling and determined the amount of variance a model could explain.

In the last step, we aimed to explore perceptual judgements by merging the intra- and interpersonal level, analogous to M3. Therefore, we focused on the feature that best explained the behavioural rating on both levels and additionally outperformed the remaining features in pairwise comparisons. We normalized the representational geometry and created a common feature space by averaging the corresponding RDMs (Fig. S3). Next, we investigated the relationship between the produced feature combination RDM and single-participant behavioural RDMs and tested the resulting model against all other feature RDMs in the same manner as described above.

To calculate features and perform data analysis we used the SAMI toolbox, which is available on Github and archived in Zenodo^35^.

## Results

### Emotion recognition of full body stimuli interactions

The present data revealed that overall emotion recognition was high. Anger sequences were categorized with the highest accuracy (M = 91.9%, SEM = 1.75), followed by happiness (M = 90.6%, SEM = 1.59), sadness (M = 87.63%, SEM = 1.77), and affection (M = 80.38%, SEM = 2.72). All four emotions were classified above chance level (happiness: t(30) = 41.48, p < 0.001; affection: t(30) = 20.36, p < 0.001; sadness: t(30) = 35.48, p < 0.001; anger: t(30) = 38.27, p < 0.001). For more information, see Supplementary Fig. S4.

### Feature-based discrimination between emotion categories

On the intrapersonal level, the kinematic feature *velocity* revealed a significant main effect of emotion category. Bonferroni-corrected post hoc pairwise comparisons showed significantly faster movements for happiness compared to affection and sadness as well as for anger compared to sadness. *Vertical movement* also presented a significant main effect of emotion category: happiness was associated with more vertical displacement than anger, affection, and sadness. *Volume average* was significantly higher for happiness and anger than for sadness. The same was found for *volume STD* in which happiness and anger interactions were depicted through higher variance in volume than sadness.

For the interpersonal features, we found a significant main effect for *IPD* showing that the distance between two people was smaller when affection was expressed compared to happiness and anger. Likewise, *IPD STD* revealed smaller variability while expressing sadness compared to affection.

Examining distance correlation features (relation between IPD and intrapersonal features) revealed that *IPD* was associated more strongly with *limb contraction* when expressing affection compared to anger. The distance between interacting people affected *volume* to a higher degree when showing affection compared to anger.

A further main effect of emotion category was revealed for *personal space*. *Personal space* differed significantly between affection and happiness and between sadness and anger, showing that interacting people spent significantly more time in the *personal space* of their counterpart while expressing affection. Additionally, *IPO* revealed a significant main effect of emotion category showing that actors turned more towards each other while expressing affection compared to happiness, sadness, or anger. Regarding the *motion–energy balance*, we found a significant main effect of emotion revealing a lower *motion–energy balance* for sadness and anger compared to happiness and affection. Finally, balance in the time facing each other showed a main effect of emotion category with the highest *IPO balance* being for interacting agents portraying affection compared to sadness and anger. All results of the conducted ANOVAs can be found in Table 2. For more information, see Supplementary Figs. S5, S6, and Supplementary Table S1.

**Table 2:** ANOVA of feature emotion categories.

	Happiness	Affection	Sadness	Anger
**Intrapersonal Features**				
**VEL (mm/s)** F(3,44) = 10.87, p < 0.001, η^2^ = 0.43	M = 374 SEM = 41.30	M = 204.71 SEM = 31.16	M = 124.14 SEM = 14.80	M = 335.24 SEM = 45.05
**VM (mm)** F(3,44) = 21.69, p < 0.001, η^2^ = 0.6	M = 748.63 SEM = 88.53	M = 248.46 SEM = 31.44	M = 188.81 SEM = 31.72	M = 497.18 SEM = 47.63
**VOL (m**^**3)**^ F(3, 44) = 5.66, p < 0.01, η^2^ = 0.3	M = 0.38 SEM = 0.05	M = 0.32 SEM = 0.03	M = 0.22 SEM = 0.02	M = 0.38 SEM = 0.02
**VOL STD (m**^**3)**^ F(3, 44) = 5.58, p < 0.01, η^2^ = 0.3	M = 0.1 SEM = 0.02	M = 0.07 SEM = 0.01	M = 0.04 SEM = 0.01	M = 0.11 SEM = 0.01

**Interpersonal Features**				
**IPD (mm)** F(3, 44) = 8.47, p < 0.001, η^2^ = 0.4	M = 1023 SEM = 89.65	M = 622 SEM = 38.14	M = 910 SEM = 89.38	M = 1191 SEM = 97.89
**IPD STD (mm)** F(3, 44) = 3.02, p < 0.05, η^2^ = 0.2	M = 97.99 SEM = 19.55	M = 122.34 SEM = 23.33	M = 45.90 SEM = 9.58	M = 99.12 SEM = 19.1
**DC LC (Pearson’s r)** F(3, 44) = 4.18, p < 0.05, η^2^ = 0.22	M = 0.43 SEM = 0.05	M = 0.55 SEM = 0.05	M = 0.62 SEM = 0.6	M = 0.39 SEM = 0.03
**DC VOL (Pearson’s r)** F(3,44) = 3.49, p < 0.05, η^2^ = 0.19	M = 0.42 SEM = 0.07	M = 0.49 SEM = 0.06	M = 0.53 SEM = 0.07	M = 0.28 SEM = 0.04
**PS (%)** F(3,44) = 7.49, p < 0.001, η^2^ = 0.34	M = 33.71 SEM = 11.96	M = 88.91 SEM = 6.92	M = 46.86 SEM = 13.50	M = 24.25 SEM = 7.85
**IPO (%)** F(3, 44) = 9.01, p < 0.001, η^2^ = 0.4	M = 40 SEM = 9 52	M = 79.63 SEM = 8.56	M = 32.21 SEM = 7.96	M = 25.15 SEM = 5.91
**ME BAL (AU)** F(3, 44) = 6.83, p < 0.001, η^2^ = 0.32	M = 0.93 SEM = 0.01	M = 0.90 SEM = 0.02	M = 0.78 SEM = 0.05	M = 0.76 SEM = 0.04
**IPO BAL (AU)** F(3, 44) = 8.42, p < 0.001, η^2^ = 0.36	M = 0.55 SEM = 0.13	M = 0.85 SEM = 0.08	M = 0.17 SEM = 0.1	M = 0.31 SEM = 0.11

### Feature importance for emotion classification

**Figure 2:**
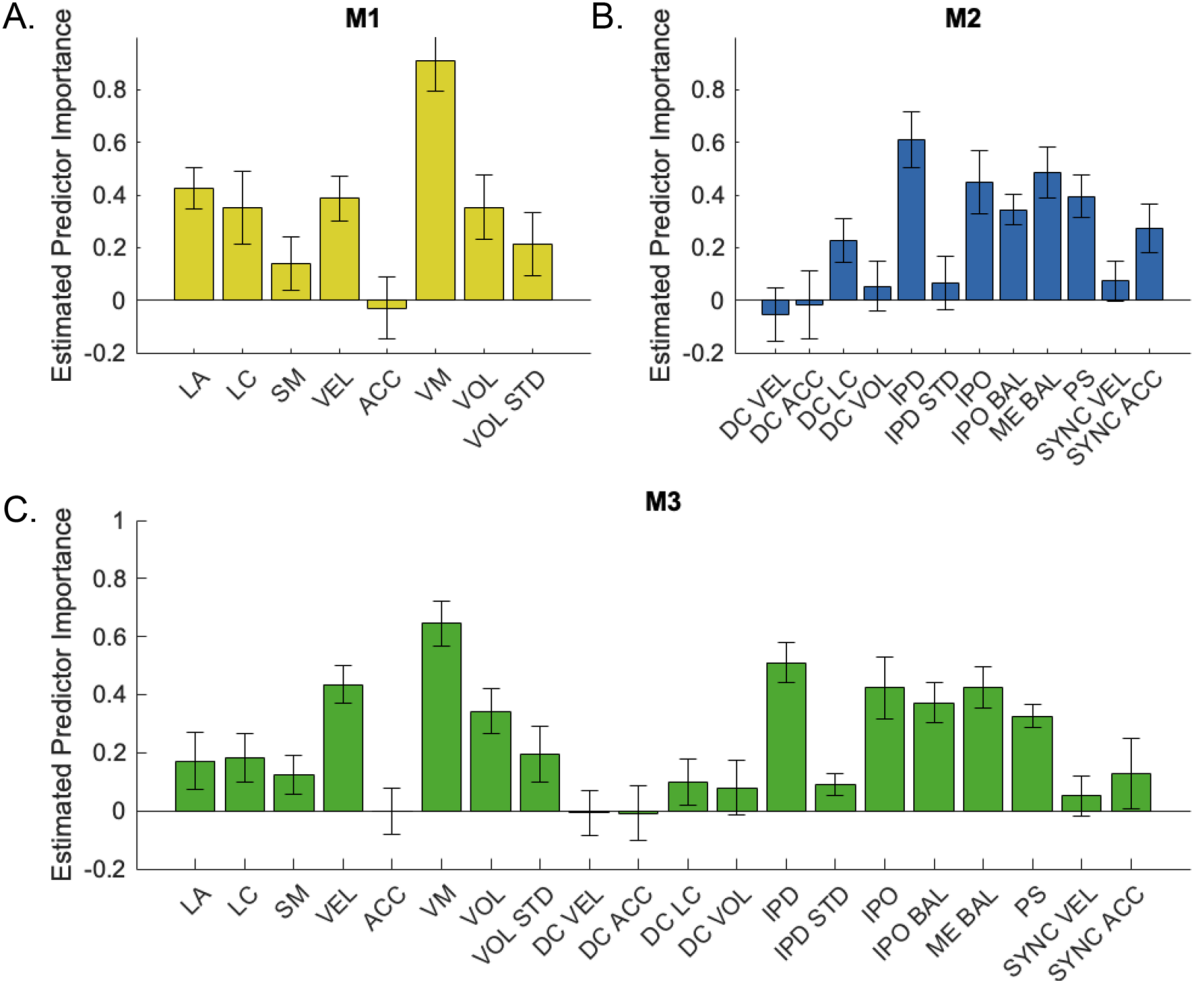
Estimated feature importance for emotion classification. (**A**) Model M1 = intrapersonal features as predictors (overall classification accuracy 56.75%). (**B**) Model M2 = interpersonal features as predictors (overall classification accuracy = 59.41%). (**C**) Model M3 = M1 + M2 combination model (overall classification accuracy of 69.54%). Bars and error bars show means and standard deviations of predictor importance for different validation samples. Chance level of emotion classification at 25%. *LA*  limb angles, *LC* limb contraction, *SM* symmetry, *VEL* velocity, *ACC* acceleration, *VM* vertical movement, *VOL* volume, *VOL* *STD* volume standard deviation, *DC* *VEL* distance correlation velocity, *DC ACC* distance correlation acceleration, *DC LC* distance correlation limb contraction, *DC* *VOL* distance correlation volume, *IPD* interpersonal distance, *IPD* *STD* interpersonal distance standard deviation, *IPO* interpersonal orientation, *IPO* *BAL* interpersonal orientation balance, *ME BAL *motion energy balance, *PS* personal space, *SYNC* *VEL* synchronization velocity, *SYNC ACC* synchronization acceleration.

To examine the relative importance of specific features for emotion classification, we trained and tested three decision tree classifiers. The models differed in terms of the features used as predictors. Model M1 (intrapersonal features) provided an overall classification accuracy of M = 62.50% (happiness: M = 50.00%, affection: M = 50.00%; sadness: M = 75.00%; anger: M = 75.00%) compared to M = 68.75% for Model M2 (interpersonal features; happiness: M = 58.33%, affection: M = 83.33%; sadness: M = 66.67%; anger: M = 66.67%). Highest overall classification accuracy was provided by the combined Model M3 with an overall classification accuracy of M = 79.17% (happiness: M = 66.67%, affection: M = 91.67%; sadness: M = 91.67%; anger: M = 66.67%). M1 revealed the highest predictor importance for *vertical movement* and *limb angles* on an intrapersonal level (Fig. 2A). M2 showed that *IPD* and *motion–energy balance* were the most relevant features for classification on an interpersonal level (Fig. 2B). The combination model (M3) revealed the highest importance of *vertical movement*, *velocity, IPD*, IPO, and *motion–energy balance* (Fig. 2C).

### Representational similarity analysis: relatedness of perceived emotions and EBL features

**Figure 3:**
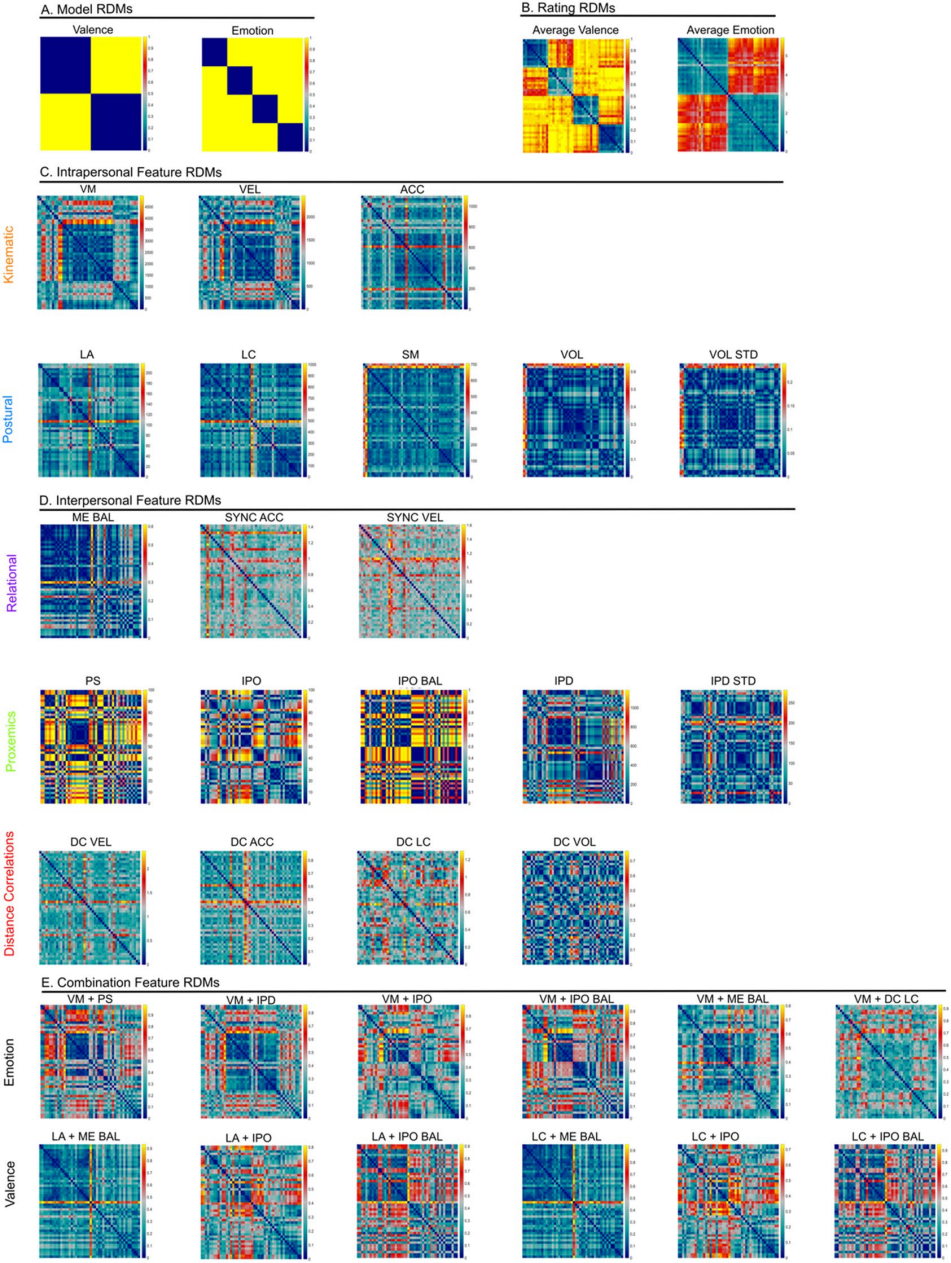
Representational dissimilarity matrices. (**A**) Theoretical model RDMs that assume different similarities based on emotion and valence categories (0 if identical, 1 otherwise). (**B**) Behavioural, averaged over participants emotion (1 if correct, 0 otherwise) and valence rating RDMs (Euclidean distance as similiarity measure). RDMs for (**C**) intrapersonal features, (**D**) interpersonal features and (**E**) combination features using Euclidean distance as similarity measure. *LA* limb angles, *LC* limb contraction, *SM* symmetry, *VEL* velocity, *ACC* acceleration, *VM* vertical movement, *VOL* volume, *VOL STD* volume standard deviation, *DC* *VEL* distance correlation velocity, *DC ACC* distance correlation acceleration, *DC L*C distance correlation limb contraction, *DC* *VOL* distance correlation volume, *IPD* interpersonal distance, *IPD* *STD* interpersonal distance standard deviation, *IPO* interpersonal orientation, *IPO* *BAL* interpersonal orientation balance, *ME BAL* motion energy balance, *PS* personal space, *SYNC* *VEL* synchronization velocity, *SYNC ACC* synchronization acceleration.

**Figure 4:**
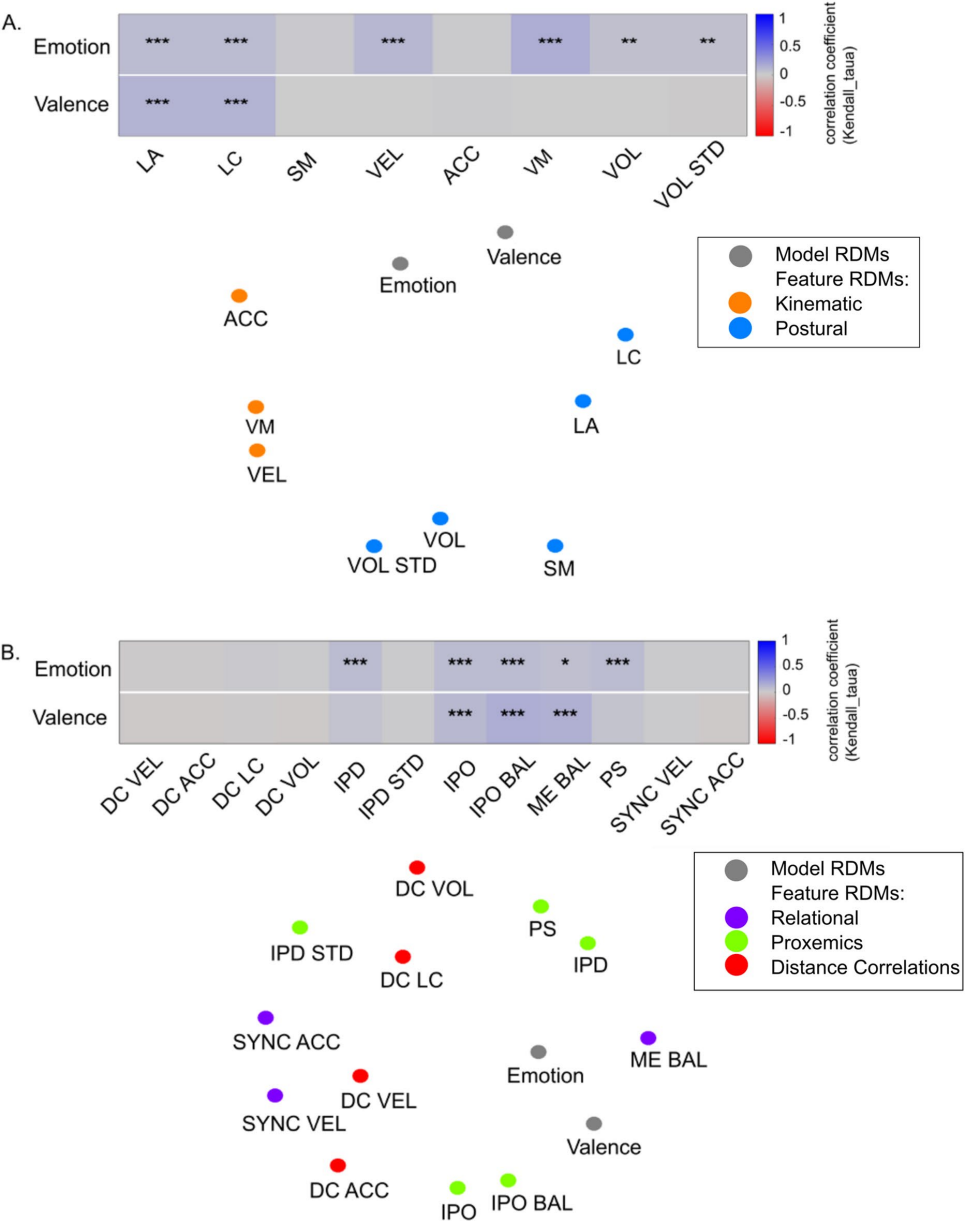
Relationship between model RDMs and feature RDMs for (**A**) intrapersonal features, (**B**) interpersonal features, as indicated by Kendall’s τ_A_ correlation. Significant correlations shown by asterisks (*ns* not significant; *p < 0.05; **p < 0.01; ***p < 0.001). MDS plots approximate Kendall’s τ_A_ correlation distance (1 − Kenadll's τA) among RDMs: the closer the points to each other, the more similar their corresponding RDMs.

To determine the relationship between the perceptual impression and EBL features, we carried out an RSA. The visual comparison between the model RDMs (Fig. 3A) and the average rating RDMs (Fig. 3B) revealed a high structural similarity. In a first step, we compared model RDMs (Fig. 3A) and feature RDMs on the intrapersonal and interpersonal levels (Fig. 3C,D). Representational distances (computed as “1 − Kendall’s τ_A_ correlation”) of the categorical and feature RDMs are depicted via MDS plots. Visual inspection of the intrapersonal MDS plot (Fig. 4A) showed a clear separation between kinematic and postural features. Within the interpersonal RDMs (Fig. 4B) *motion–energy balance* was located closest to emotion and valence category RDMs.

Feature RDMs of *vertical movement, velocity, limb angles, limb contraction,* and *volume & volume STD* correlated positively with the emotion category model RDM. *Limb angles* and *limb contraction* also correlated positively with the valence model RDM. Regarding interpersonal features, we found weak positive correlations between *IPO balance*, *IPD*, *personal space*, *IPO*, and *motion–energy balance* and the emotion category model RDM; as well as between *IPO Balance*, *motion–energy balance*, and *IPD* and the valence model RDM (Fig. 4A,B).

**Figure 5:**
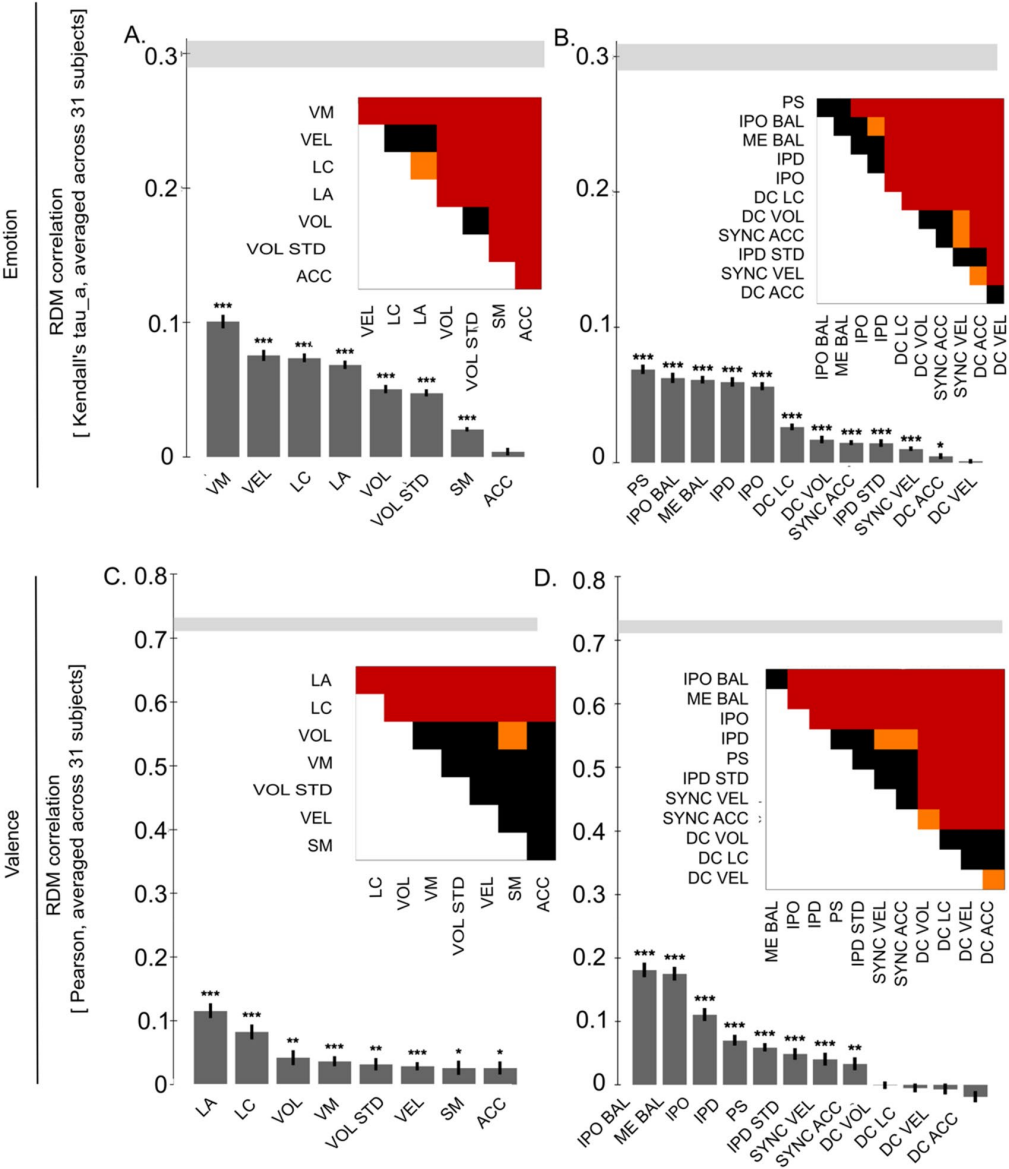
Relationship between behavioural RDMs and feature RDMs. (**A**,**C**) Intrapersonal Features; (**B**,**D**) Interpersonal Features; Kendall's τ_A_ correlation between emotion rating RDMs and Pearson correlation between valence rating RDMs. Significant correlations shown by asterisks (*ns* not significant; *p < 0.05; **p < 0.01; ***p < 0.001, controlling FDR at 0.05). Lower and upper bounds of the noise ceiling are depicted by a grey bar. Pairwise comparisons indicate which feature RDMs perform significantly differently. Colour corresponds to significance level (black: ns; orange: p < 0.05; red: p < 0.01; calculated via two-sided signed-rank test across subjects, controlling FDR at 0.05). *LA* limb angles, *LC* limb contraction, *SM* symmetry, *VEL* velocity, *ACC* acceleration, *VM* vertical movement, *VOL* volume, *VOL* *STD* volume standard deviation, *DC VEL* distance correlation velocity, *DC ACC* distance correlation acceleration, *DC* *LC* distance correlation limb contraction, *DC VOL* distance correlation volume, *IPD* interpersonal distance, *IPD* *STD* interpersonal distance standard deviation, *IPO* interpersonal orientation, *IPO* *BAL* interpersonal orientation balance, *ME BAL* motion energy balance, *PS* personal space, *SYNC* *VEL* synchornization velocity, *SYNC ACC* synchronization acceleration.

Second, we determined the relatedness between EBL features and perceptual impressions by correlating emotion- and valence-rating RDMs and intra- and interpersonal model RDMs. Regarding the relationship between perceived emotion and intrapersonal features, we found significant correlations for all kinematic and postural parameters except *acceleration* (Fig. 5A). The highest correlations were for *vertical movement* (r = 0.1) and *velocity* (r = 0.08). It has to be noted that all correlations were rather low ranging from 0.01 to 0.1. Nevertheless, it is worth mentioning that *vertical movement* performed better than the remaining features as revealed by pairwise comparisons between the feature RDMs (Fig. 5A). None of the feature RDMs came close to the noise ceiling (0.29–0.31).

When comparing intrapersonal features with valence ratings, we identified significant correlations for each kinematic and postural feature ranging from 0.03 to 0.14. Data revealed that postural parameters performed better than kinematic parameters. As revealed by pairwise comparisons, *limb angles* correlated most strongly (r = 0.12) with valence ratings and performed significantly better than all other models (Fig. 5C). The second strongest correlation (r = 0.08) was found for *limb contraction,* which additionally outperformed all kinematic features. Hence, kinematic intrapersonal EBL features related more strongly to the perceived emotion category, and postural intrapersonal EBL features related more strongly to perceived valence.

The comparison between interpersonal feature RDMs and emotion category rating RDMs (Fig. 5B) revealed the highest correlation for *personal space* (r = 0.07). Furthermore, *IPO balance* (r = 0.06) and *motion–energy balance* (r = 0.06), as well as *IPD* (r = 0.06), *IPO* (r = 0.06), and *distance correlation limb contraction* (r = 0.03) performed significantly better than the remaining models (p < 0.001).

Regarding the comparison between interpersonal features and valence ratings (Fig. 5D), the highest explanatory value was provided by *IPO balance* (r = 0.18). This also outperformed all other models (p < 0.001) with the exception of *motion–energy balance* (r = 0.18). Except for the four *distance correlation* RDMs, all interpersonal features attained weak significant correlations with valence ratings. Thus, emotion and valence perception of interacting people seems to depend most strongly on the displayed *motion–energy balance* and orientation as well as on proxemic measures (*IPD*, *IPO*, *personal space*).

Furthermore, we conducted an explorative analysis of feature combinations (Fig. 3E). Regarding emotion perception, we averaged vertical movement with each of the six highest performing interpersonal features (*IPO balance, personal space, motion–energy balance, IPO, IPD, DC LC*). Only feature combinations between *vertical movement* and *IPO* (r = 0.11) as well as between *vertical movement* and *motion–energy balance* (r = 0.11) performed significantly better than the remaining combination models and all intra- and interpersonal models (p < 0.001) except for the combination between *vertical movement* and *IPO balance.* This indicates that emotion perception of EBL was best predicted not by a single feature in isolation, but by a combination of several features.

Regarding valence perception, averaging *limb angles* and *IPO balance* (r = 0.21), as well as *limb contraction* and *IPO balance* (r = 0.2) revealed higher correlations. Furthermore, pairwise comparisons revealed significant differences between all combination RDMs and feature RDMs on both levels (p < 0.001), except for the combination between limb angles and *motion–energy balance* as well as the single feature *motion–energy balance*. For more information, see Supplementary Figs. S9, S10 and Supplementary Table 2.

## Discussion

Our data provide a detailed quantitative description of movement features in emotional interactions that are related to emotion perception. The systematic decomposition of an interaction into an intrapersonal and interpersonal level reveals that both levels relate substantially to the emotional content of the scene as well as to its perception. We show that the emotional content of social interactions has a specific kinematic and postural fingerprint and can be described via quantitative intra- and interpersonal parameters. Both levels are linked to each other inseparably. This linkage is reflected not only by a model that integrates intra- and interpersonal features (M3) exhibiting the best performance but also by the explorative analysis of feature combinations. We further show a strong correspondence between those features that characterize the emotional content of a stimulus and the features that are critical for emotion perception^3^. Representational similarity analysis reveals that it is especially kinematic parameters that contribute to the perception of emotional content on an intrapersonal level; whereas on an interpersonal level, balance and proxemics parameters are important cues for the observer. It also becomes apparent that observers use mainly interaction-specific information to decode relational emotions such as affection. We further found that intrapersonal postural parameters such as *limb angles* and interpersonal balance parameters such as *motion–energy balance* and *IPO balance* show the strongest relation to the valence percept.

Recently, de Gelder and Poyo Solanas have proposed a framework in which perceptually relevant information from bodies via movement and posture is coded in the brain through midlevel features such as limb contraction and head-to-hand distance^43^. Our results support the importance of these midlevel features and add computational interaction-specific parameters to their framework. The present data show that the emotional content of a scene is characterized by midlevel features such as *velocity* or *motion–energy balance*. For example, happy interactions are characterized by higher *velocity* profiles than affection and sadness, but not higher than anger. These findings are broadly consistent with those reported in the existing literature^2,3,19,20,22^. Affectional and sad interactions show a high degree of similarity regarding their intrapersonal kinematic and postural parameters. These emotions, however, reveal characteristic differences on the interpersonal level (e.g., *IPO, IPO Balance, IPD STD, personal space*).

Regarding emotion perception, our findings show an association to characteristic body expressions on both the intra- and the interpersonal level. Representational similarity analysis reveals that *vertical movement*, *IPO (average & orientation)*, and *motion–energy balance* are best suited to explain emotion perception*.* In contrast to some research reports^12,24,36,44^, we were unable to distinguish emotional categories via postural features such as *limb angles* and *limb contraction*. Here, it has to be taken into account that most former studies used stimuli depicting a single person mainly in a frontal view and not social interactions observed from a third-person perspective as in the present study. The present data show that participants confused happiness with anger, although only to a small extent. Conversely, anger trials were more often confused with sadness than with happiness. Most often, affection stimuli were confused with happiness. A study investigating emotions in gait^3^ has demonstrated that confusions occur preferentially between emotions that share a similar level of movement activation: angry gaits tend to be confused with happy gaits, and sad gaits with fearful ones. Thus, these authors concluded that *velocity* is particularly important for the perception and expression of emotions^3,20,22^. Our findings also suggest that velocity of movements is important in the process of emotion recognition. However, velocity is not sufficient to distinguish between emotions such as anger and happiness, especially within social interactions where interpersonal cues such as proxemics or balance are available for the observer. Interpersonal cues such as *motion–energy balance* between two agents allow a perceptual distinction between happiness and anger. *Motion-energy balance* explains (1) the high degree of confusion between happiness and affection and (2) the low degree of confusion between anger and happiness when social information is available. *Motion-energy balance* within interactions, therefore, seems to be an important property for the observer to generate an emotional percept. Hence, social context information is particularly important for recognizing emotional content, especially when the depicted emotions depend more on reciprocal interactions (e.g., affection)^10,45^. The present results provide a computational framework for this observation. For example, affection differs from other emotions only regarding its interpersonal movement characteristics. This is underpinned by the calculated classification trees: the intrapersonal model is less accurate than the interpersonal model, underlining that emotions such as affection have a strong interpersonal character and that the spatiotemporal coupling of two moving agents seems to be of great significance especially for perceiving socially expressible emotions^10,17^.

Besides emotion recognition, we were interested in the perceived emotional valence—a dimension that reflects the subjective impression of a scene related to approach–avoidance tendencies^46^. Our data reveal that on the intrapersonal level, postural features such as limb angles best explain the participants’ valence perception. Regarding interpersonal features, *motion–energy balance* and orientation between interacting people are the best predictors of perceived valence.

Finally, we observed a noteworthy, albeit not significant, trend towards a synchronization of *velocity* profiles, indicating that higher synchronization between people is associated with a positive impression of the perceived interaction. A study investigating interpersonal behaviour in a social task has shown that patterns of proxemic behaviours and interpersonal distance predicted the subjective quality of interactions^28^. Thus, balance and spatiotemporal harmony are predictors for both the experienced and the observed quality of an interaction.

Interestingly, our RSA results show that emotion category recognition is better predicted by kinematic features, whereas valence perception is related more to postural features of the stimuli. Basically, human emotions can be conceptualized within a two-dimensional model comprised of emotional valence (the subjective value—i.e., positive vs negative) and arousal (intensity)^47,48^. The present results reveal that emotions possessing the same valence (e.g., anger and sadness) are more similar in terms of the actors' postural features. Further, we observed that emotions that differ in terms of their valence but are similar in terms of their intensity (e.g. happiness and anger) resemble each other regarding their kinematics. Thus, one might assume that postural features might be more likely to reflect the valence and kinematic features might be more likely to reflect the arousal or intensity of the presented stimuli.

Altogether, we found a set of EBL features that characterizes emotional content and predicts the perception of the emotional quality of human interactions. These features are defined on an intra- and interpersonal level and include kinematic, and postural characteristics as well as proximity, balance, and synchronization. We conclude that the perception of human emotional interactions is a function of not only inherent kinematics of the agent but also interpersonal balance and proximity between agents.

### Limitations and future implications

It should be noted that the present and comparable studies differ with respect to the stimulus material used, stimulus length, emotional content, contextual information, and feature calculation^17,24^. These differences explain the partly heterogeneous results on emotion perception. Despite this heterogeneity, perception and recognition of emotional content are robust regardless of the stimulus material used. Thus, humans seem to weigh the relative importance of different movement features flexibly depending on the specific stimulus properties presented to them.

We have to acknowledge that neither an intrapersonal nor an interpersonal feature correlates with the perceptual performance on the noise ceiling level, and that we found only weak positive correlations in the present study^24^. One reason for this may be that many features are similarly pronounced in different emotion categories. For example, happiness and anger are characterized by similar velocities. Hence, it would seem appropriate to develop models that integrate multiple feature dimensions of the observed scene. First solutions are offered by the present attempt to use a combination of features to classify the emotional content as well as to predict the emotional percept. Future studies, however, might apply more ecologically valid stimuli and combine different features in a multidimensional space in order to phenotype emotion specific properties of EBL in social interactions. Such approaches that aim to decode emotional human states from a combination of nonverbal signals on multiple levels are highly relevant in the context of human–robot interaction in order to ensure natural communication^47–50^.

## Supplementary Information

Supplementary Information.

## Data Availability

The datasets used and analyzed during the current study are available from the corresponding author on reasonable request. The source code is available at https://zenodo.org/record/4764552#.YiXYKi9Xb0p (MATLAB).

## References

[1] Atkinson AP, Dittrich WH, Gemmell AJ, Young AW (2004). Emotion perception from dynamic and static body expressions in point-light and full-light displays. Perception.

[2] Michalak J, Troje NF, Fischer J, Vollmar P, Heidenreich T, Schulte D (2009). Embodiment of sadness and depression—gait patterns associated with dysphoric mood. Psychosom. Med..

[3] Roether CL, Omlor L, Christensen A, Giese MA (2009). Critical features for the perception of emotion from gait. J. Vis..

[4] Bänziger T, Grandjean D, Scherer KR (2009). Emotion recognition from expressions in face, voice, and body: The Multimodal Emotion Recognition Test (MERT). Emotion.

[5] Derntl B, Habel U (2011). Deficits in social cognition: A marker for psychiatric disorders?. Eur. Arch. Psychiatry Clin. Neurosci..

[6] Frith CD, Frith U (2012). Mechanisms of social cognition. Annu. Rev. Psychol..

[7] Lorey B, Kaletsch M, Pilgramm S, Bischoff M, Kindermann S, Sauerbier I, Stark R, Zentgraf K, Munzert J (2012). Confidence in emotion perception in point-light displays varies with the ability to perceive own emotions. PLoS One.

[8] Kaletsch M, Pilgramm S, Bischoff M, Kindermann S, Sauerbier I, Stark R, Lis S, Gallhofer B, Sammer G, Zentgraf K, Munzert J, Lorey B (2014). Major depressive disorder alters perception of emotional body movements. Front. Psychiatry..

[9] Kleinsmith A, Bianchi-Berthouze N (2013). Affective body expression perception and recognition: A survey. IEEE Trans. Affect. Comput..

[10] Clarke TJ, Bradshaw MF, Field DT, Hampson SE, Rose D (2005). The perception of emotion from body movement in point-light displays of interpersonal dialogue. Perception.

[11] de Gelder B (2006). (2006) Towards the neurobiology of emotional body language. Nat. Rev. Neurosci..

[12] Atkinson AP, Tunstall ML, Dittrich WH (2007). Evidence for distinct contributions of form and motion information to the recognition of emotions from body gestures. Cognition.

[13] de Gelder B (2009). (2009) Why bodies? Twelve reasons for including bodily expressions in affective neuroscience. Philos. Trans. R. Soc. B..

[14] Aviezer H, Trope Y, Todorov A (2012). Body cues, not facial expressions, discriminate between intense positive and negative emotions. Science.

[15] Goldberg H, Christensen A, Flash T, Giese MA, Malach R (2015). Brain activity correlates with emotional perception induced by dynamic avatars. Neuroimage.

[16] Bachmann J, Munzert J, Krüger B (2018). Neural underpinnings of the perception of emotional states derived from biological human motion: A review of neuroimaging research. Front. Psychol..

[17] Bachmann J, Zabicki A, Munzert J, Krüger B (2020). Emotional expressivity of the observer mediates recognition of affective states from human body movements. Cogn. Emot..

[18] Ekman P, Friesen WV (1971). Constants across cultures in the face and emotion. J. Pers. Soc. Psychol..

[19] Barliya A, Omlor L, Giese MA, Berthoz A, Flash T (2012). Expression of emotion in the kinematics of locomotion. Exp. Brain Res..

[20] Wallbott HG (1998). Bodily expression of emotion. Eur. J. Soc. Psychol..

[21] Paterson, H. M., Pollick, F. E., & Sanford, A. J. (2001) The role of velocity in affect discrimination: 6.

[22] Pollick FE, Paterson HM, Bruderlin A, Sanford AJ (2001). Perceiving affect from arm movement. Cognition.

[23] Glowinski D, Dael N, Camurri A, Volpe G, Mortillaro M, Scherer K (2011). Toward a minimal representation of affective gestures. IEEE Trans. Affective Comput..

[24] Poyo Solanas M, Vaessen MJ, de Gelder B (2020). The role of computational and subjective features in emotional body expressions. Sci. Rep..

[25] Gross MM, Crane EA, Fredrickson BL (2012). Effort-Shape and kinematic assessment of bodily expression of emotion during gait. Hum. Mov. Sci..

[26] Van den Stock J, Righart R, de Gelder B (2007). Body expressions influence recognition of emotions in the face and voice. Emotion.

[27] Moreau Q, Galvan L, Nazir TA, Paulignan Y (2016). Dynamics of social interaction: Kinematic analysis of a joint action. Front. Psychol.

[28] Lahnakoski JM, Forbes PAG, McCall C, Schilbach L (2020). Unobtrusive tracking of interpersonal orienting and distance predicts the subjective quality of social interactions. R. Soc Open Sci..

[29] Yokozuka T, Ono E, Inoue Y, Ogawa K-I, Miyake Y (2018). The relationship between head motion synchronization and empathy in unidirectional face-to-face communication. Front. Psychol..

[30] Troje NF, Westhoff C, Lavrov M (2005). Person identification from biological motion: Effects of structural and kinematic cues. Percept. Psychophys..

[31] Overhill H (2014). Apple pie proxemics: Edward T. Hall in the kitchen work triangle. Des. Issues.

[32] Sorokowska A, Sorokowski P, Hilpert P (2017). Preferred interpersonal distances: A global comparison. J. Cross Cult. Psychol..

[33] Thepsoonthorn C, Yokozuka T, Miura S, Ogawa K, Miyake Y (2016). Prior knowledge facilitates mutual gaze convergence and head nodding synchrony in face-to-face communication. Sci. Rep..

[34] Thurman S, Lu H (2014). Perception of social interactions for spatially scrambled biological motion. PLoS One.

[35] Zabicki, A. & Keck, J. (2021) SAMI: Similarity Analysis of Human Movements and Interactions (Version v0.1.0). Zenodo 10.5281/zenodo.4764552.

[36] Aronoff J, WoikeHyman BALM (1992). Which are the stimuli in facial displays of anger and happiness? Configurational bases of emotion recognition. J. Pers. Soc. Psychol..

[37] Silver NC, Dunlap WP (1987). Averaging correlation coefficients: Should fisher’s z transformation be used?. J. Appl. Psychol..

[38] Berry KJ, Mielke PW (2000). A Monte Carlo Investigation of the Fisher Z transformation for normal and nonnormal distributions. Psychol. Rep..

[39] Opitz D, Maclin R (1999). Popular ensemble methods: An empirical study. J. Artif. Intell. Res..

[40] Loh W-Y (2002). Regression tress with unbiased variable selection and interaction detection. Stat. Sin.

[41] Kriegeskorte N, Mur M, Ruff DA, Kiani R, Bodurka J, Esteky H, Tanaka K, Badettini PA (2008). Matching categorical object representations in inferior temporal cortex of man and monkey. Neuron.

[42] Nili H, Wingfield C, Walther A, Su L, Marslen-Wilson W, Kriegeskorte N (2014). A toolbox for representational similarity analysis. PLoS Comput. Biol..

[43] de Gelder B, Poyo SM (2021). A computational neuroethology perspective on body and expression perception. Trends Cogn. Sci..

[44] Dittrich WH, Troscianko T, Lea SEG, Morgan D (1996). Perception of emotion from dynamic point-light displays represented in dance. Perception.

[45] Kret ME, de Gelder B (2010). Social context influences recognition of bodily expressions. Exp. Brain Res..

[46] Frijda NH (1986). The Emotions.

[47] Sapiński T, Kamińska D, Pelikant A, Anbarjafari G (2019). Emotion recognition from skeletal movements. Entropy.

[48] Noroozi F, Corneanu CA, Kamińska D, Sapiński T, Escalera S, Anbarjafari G (2021). Survey on emotional body gesture recognition. IEEE Trans. Affect. Comput..

[49] Wang S, Li J, Cao T, Wang H, Tu P, Li Y (2020). Dance emotion recognition based on laban motion analysis using convolutional neural network and long short-term memory. IEEE Access..

[50] Zacharatos H, Gatzoulis C, Charalambous P, Chrysanthou Y (2021). Emotion recognition from 3D motion capture data using deep CNNs. IEEE Conf. Games.

