# Supplementary material for: Decoding spatiotemporal features of emotional body language in social interactions
Source: Sci Rep. 2022 Sep 5;12:15088. doi: 10.1038/s41598-022-19267-5 (PMC9445068; doi:10.1038/s41598-022-19267-5)
Supplement: Supplementary file 1 — Supplementary Information. [file 41598_2022_19267_MOESM1_ESM.docx]

**Supplementary Information**

**
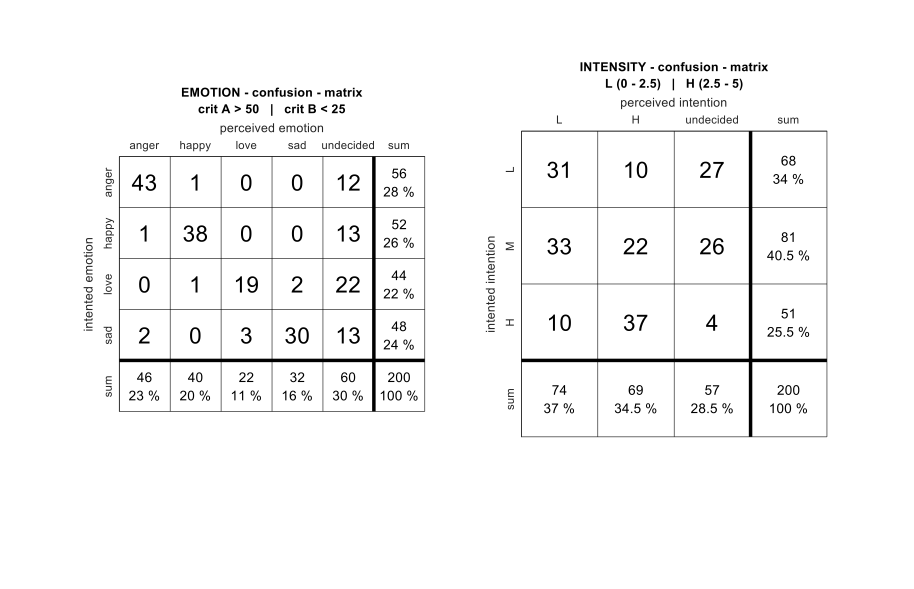
**

**S1 Fig. Validation.** Prior Validation confusion matrix which was used as base for the stimulus selection

####

| Emotion | Fold | 1 | 2 | 3 | 4 | 5 | 6 | 7 | 8 | 9 | 10 | 11 | 12 |
| --- | --- | --- | --- | --- | --- | --- | --- | --- | --- | --- | --- | --- | --- |
| Happiness | 1 | Test | Train | | | | | | | | | | |
|  | 2 | Train | Test | Train | | | | | | | | | |
|  | 3 | Train | | Test | Train | | | | | | | | |
|  | : | : | : | : | : | : | : | : | : | : | : | : | : |
|  | 12 | Train | | | | | | | | | | | Test |
| Affection | 1 | Test | Train | | | | | | | | | | |
|  | 2 | Train | Test | Train | | | | | | | | | |
|  | 3 | Train | | Test | Train | | | | | | | | |
|  | : | : | : | : | : | : | : | : | : | : | : | : | : |
|  | 12 | Train | | | | | | | | | | | Test |
| Sadness | 1 | Test | Train | | | | | | | | | | |
|  | 2 | Train | Test | Train | | | | | | | | | |
|  | 3 | Train | | Test | Train | | | | | | | | |
|  | : | : | : | : | : | : | : | : | : | : | : | : | : |
|  | 12 | Train | | | | | | | | | | | Test |
| Anger | 1 | Test | Train | | | | | | | | | | |
|  | 2 | Train | Test | Train | | | | | | | | | |
|  | 3 | Train | | Test | Train | | | | | | | | |
|  | : | : | : | : | : | : | : | : | : | : | : | : | : |
|  | 12 | Train | | | | | | | | | | | Test |

**S2 Fig. Emotion classification with decision trees.** We used Leave-One-Out Cross Validation to evaluate the goodness or our models. Each stimulus was used as test set while the remaining stimuli worked as training data. To avoid imbalanced datasets and therefore bias each category was presented equally in training and test data.


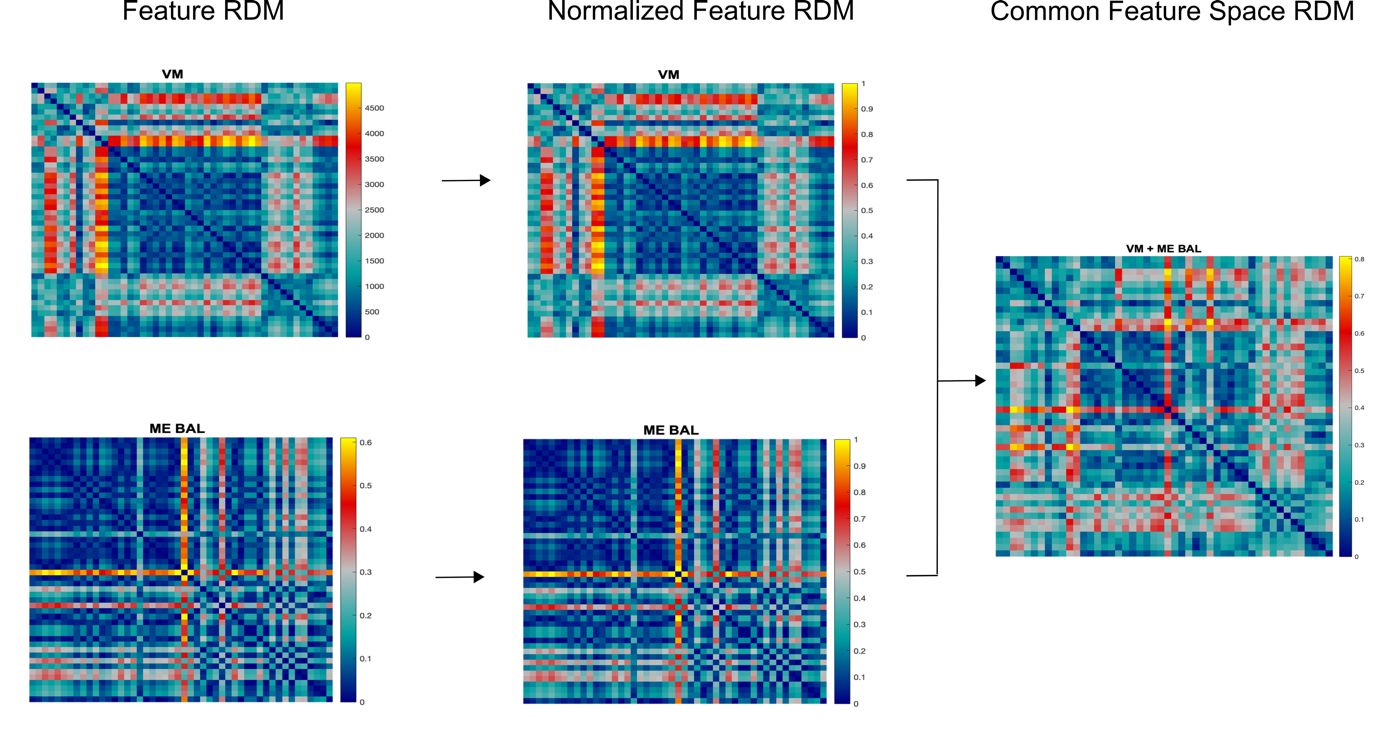


**S3 Fig. Creating a Common Feature Space.** Normalization of distance measure (Euclidean distances) into a range of values between 0 and 1 by using minima and maxima distances of the feature RDMs. Afterwards, averaging of the normalized feature RDMS to create a common feature space.

**
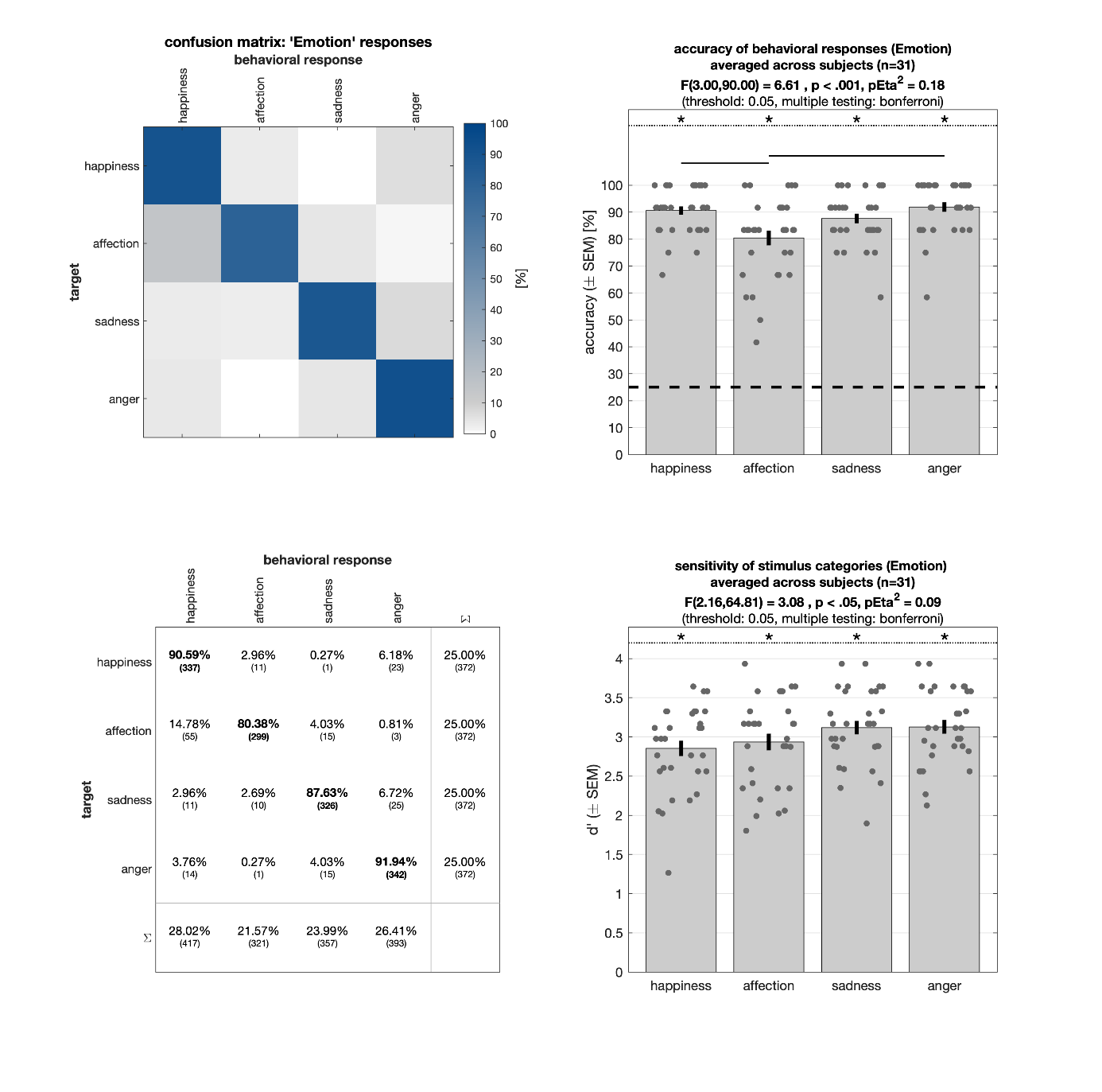
**

**S4 Fig. Emotion Recognition Rate, sensitivity and emotion dependent differences.** In summary, emotion recognition was high for each emotion category. Among the emotions, anger was most recognizable (M = 91.9%), followed by happiness (M = 90.6%), sadness (M = 87.6%) and affection (80.4%). Dots represent the distribution of individual performances regarding emotion recognition and sensitivity. Affection stimuli were most often confused with happiness (14.78 %). However, recognition performance was within a fully satisfying range, which underlines the validity of the stimuli in the context of the present study. High within-category similarity was found for happiness (SEM = 1.59), sadness (SEM = 1.77) and anger (SEM = 1.75) indicated by error bars. Lowest within category similarity was found for affection recognition (SEM = 2.72). The validity is also underlined as there are no significant differences in the sensitivity of the categories analysed by a repeated measures ANOVA (F(3,72) = 1.92, p = 0.13).


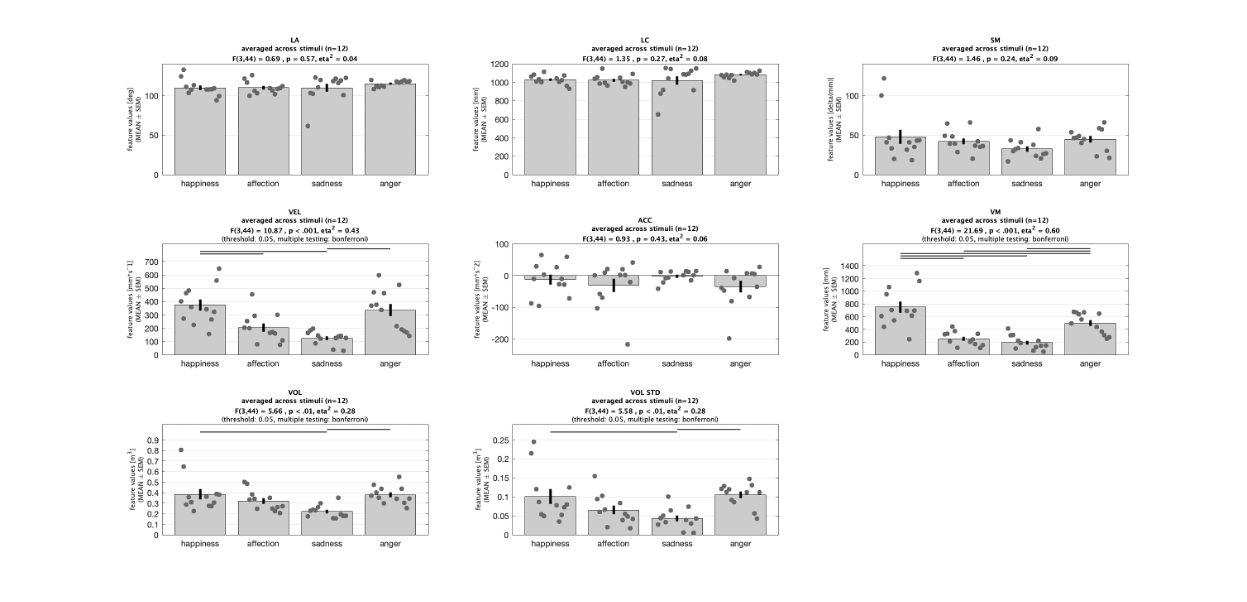


**S5 Fig.** **Feature differences across emotions on an intrapersonal level.** Significant feature differences and calculated values. Grey bars and their SEM showing corresponding average values. Horizonal lines indicate significant differences between emotion categories on intrapersonal features.

**S6 Fig. Feature differences across emotions on an interpersonal level.** Significant feature differences and calculated values. Grey bars and their SEM showing corresponding
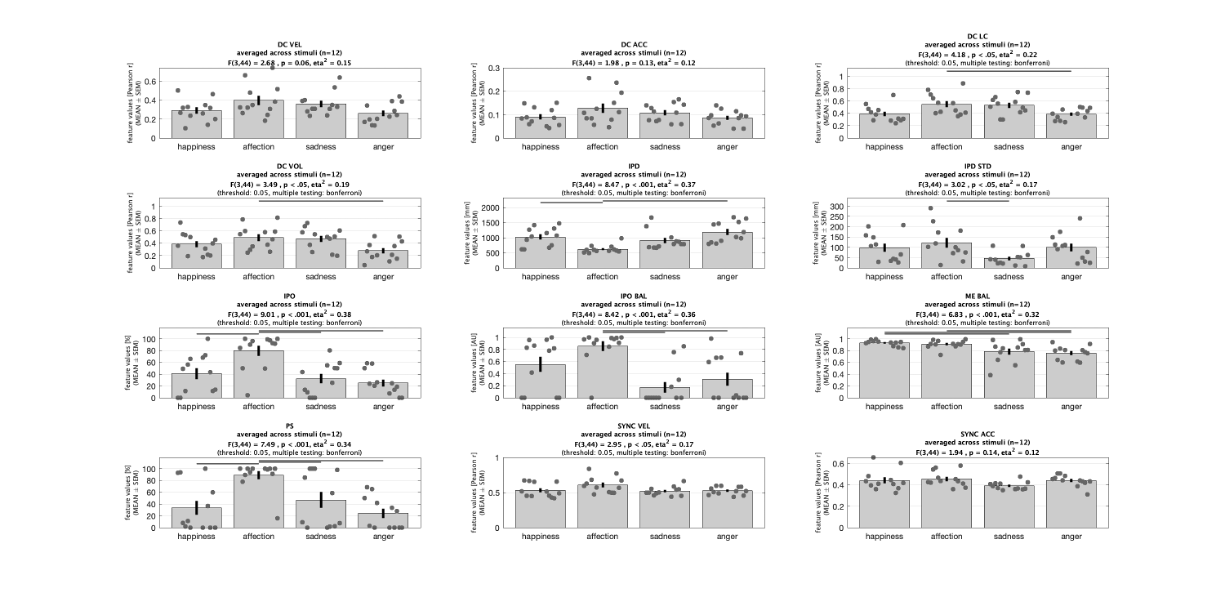
average values. Horizonal lines indicate significant differences between emotion categories on interpersonal features.

**
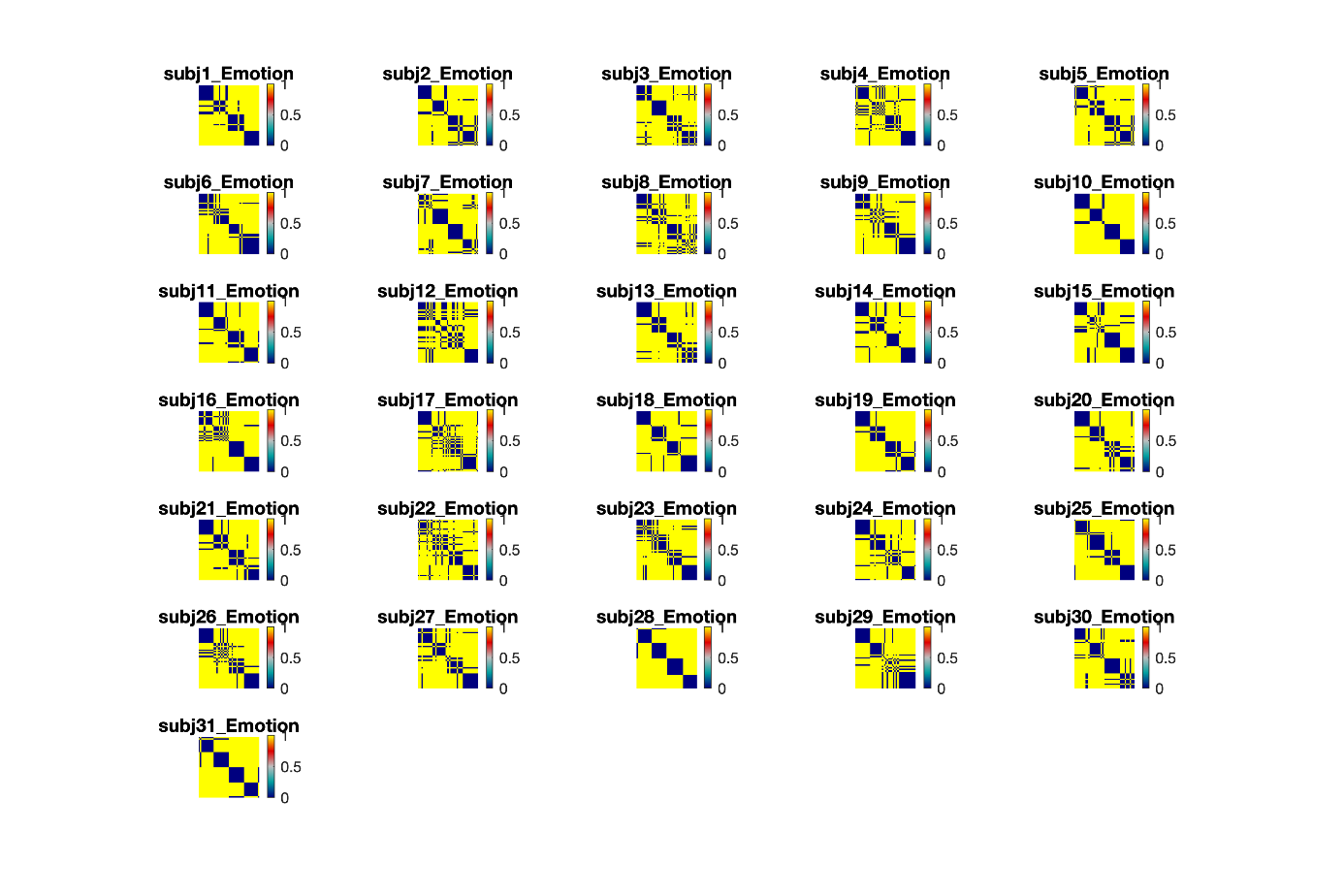
**

**S7 Fig. Behavioural emotion rating RDMs.** RDMs of emotion ratings for each participant


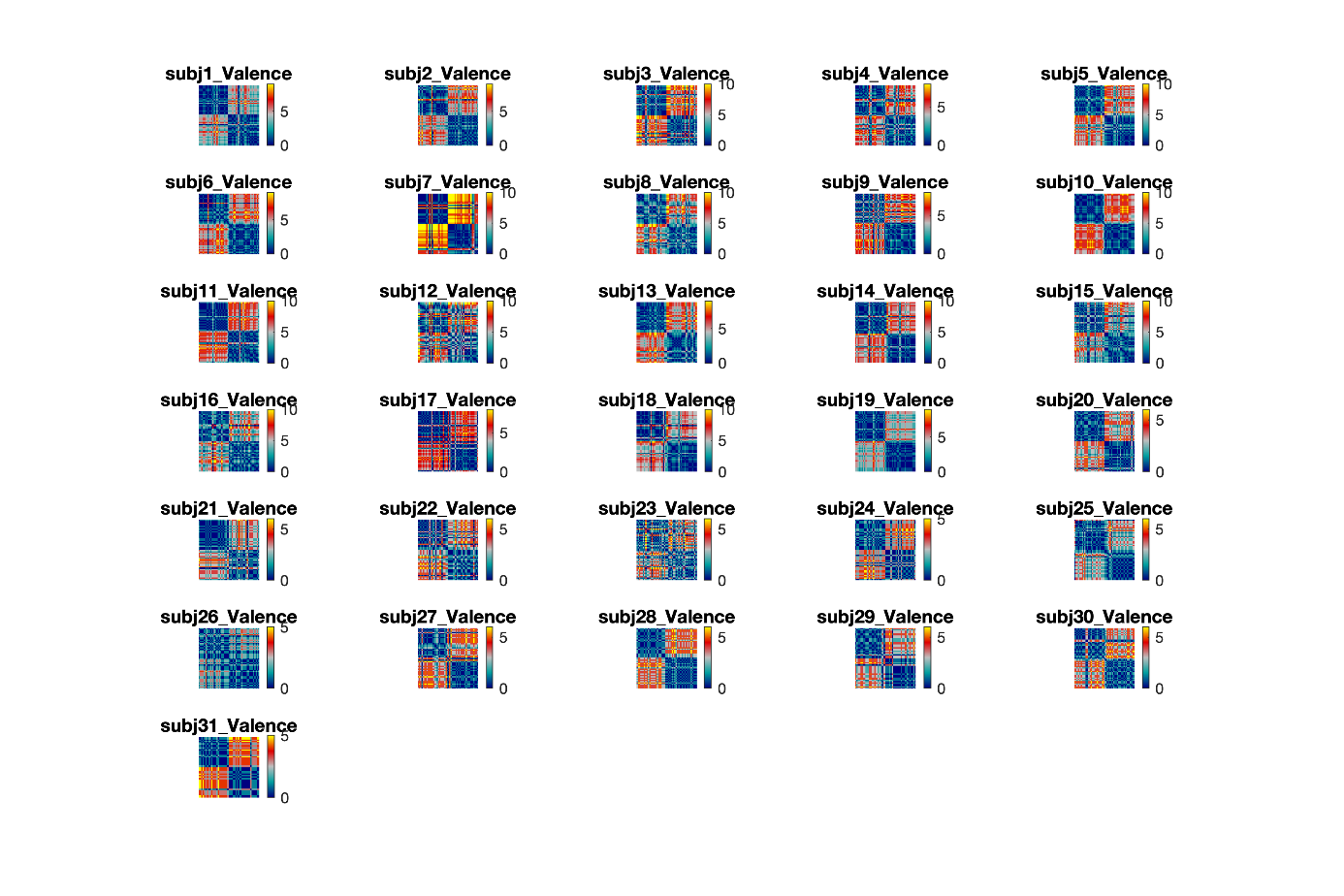


**S8 Fig. Behavioural valence rating RDMs**. RDMs of valence ratings for each participant


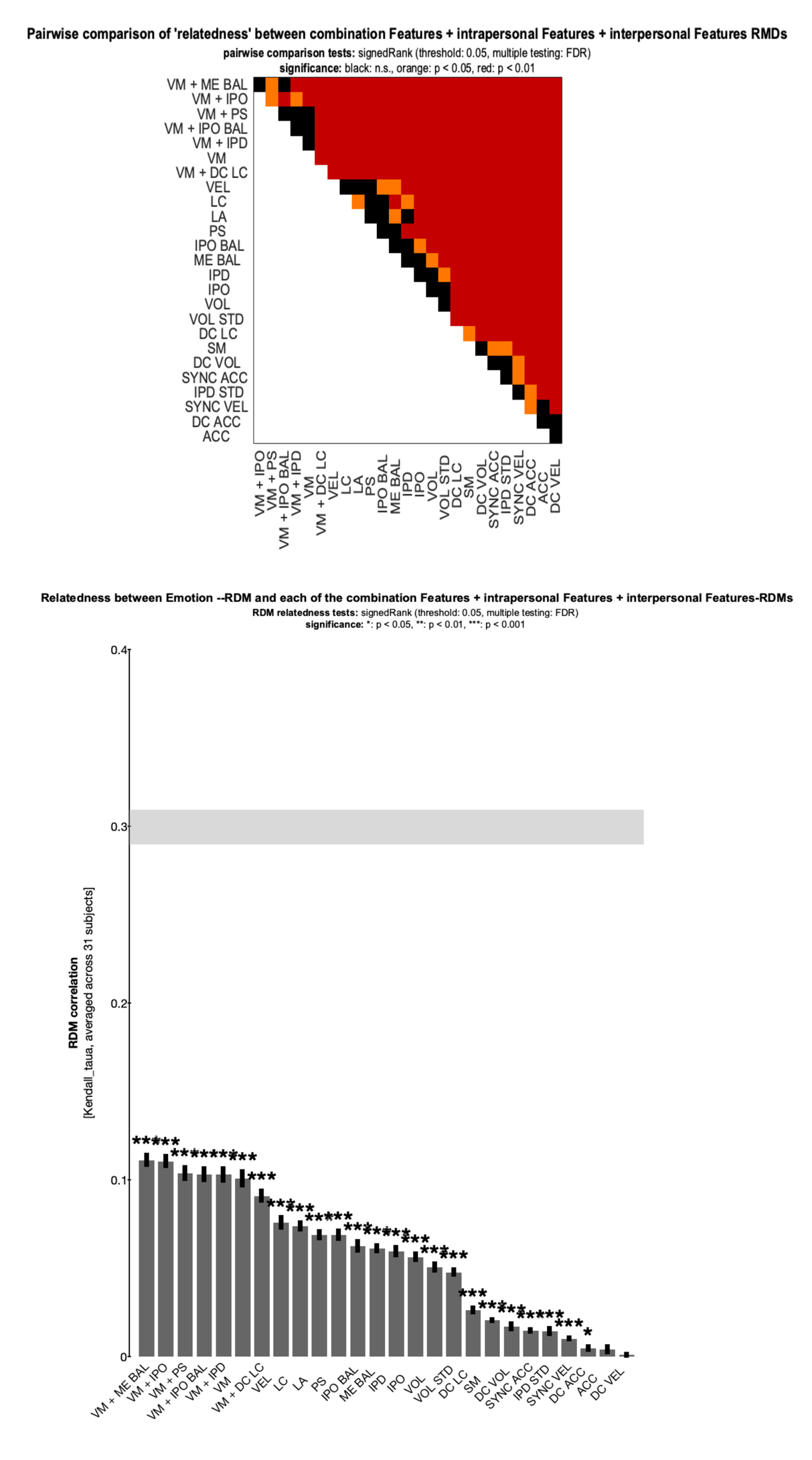


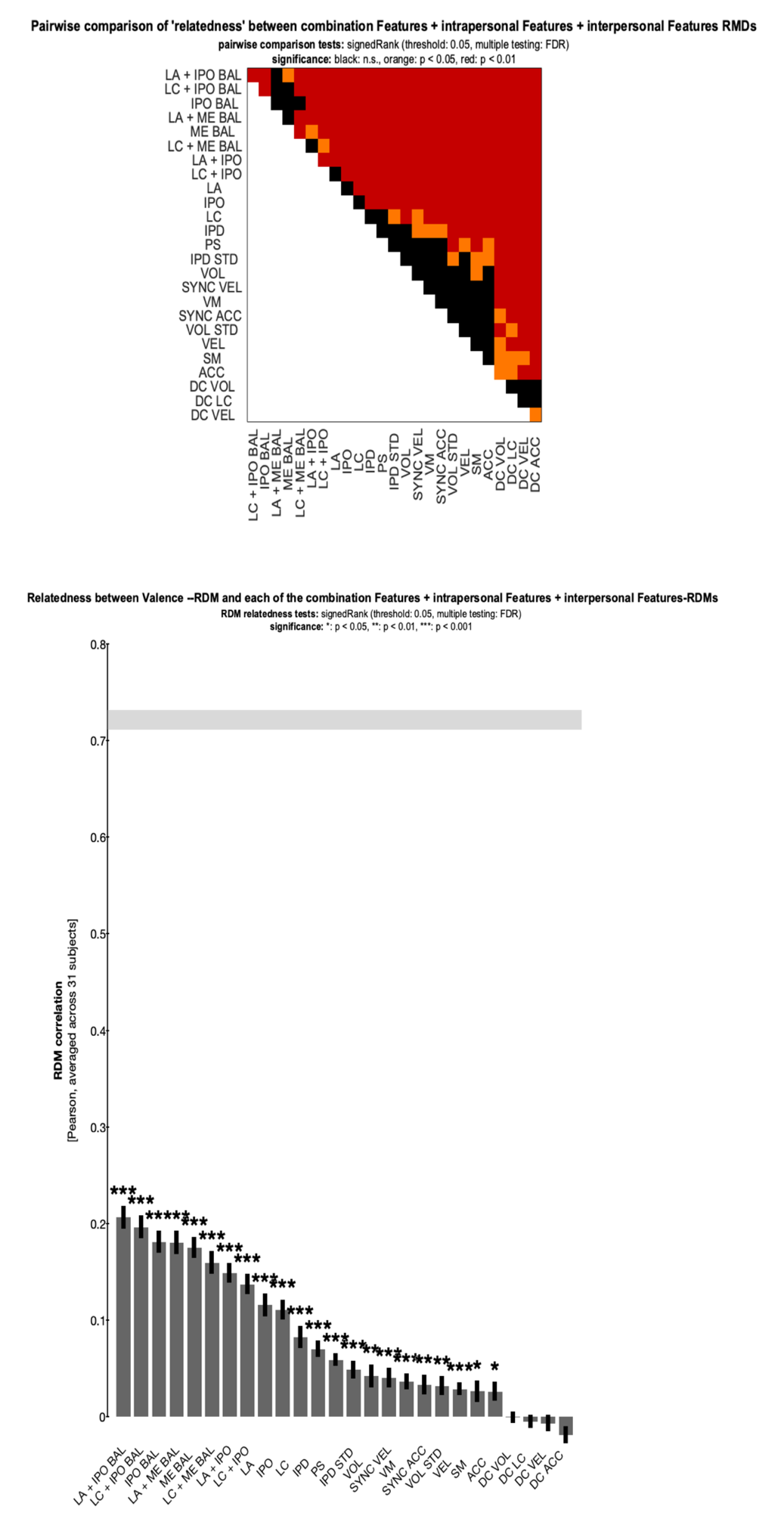


**S 9 & S10 Fig.** Relationship between behavioural RDMs and feature combination RDMs

| **S1 Table. ANOVA of Feature Emotion Categories**  **Intrapersonal** | | | | | | |
| --- | --- | --- | --- | --- | --- | --- |
| **Cases** | **Sum of Squares** | **df** | **Mean Square** | **F** | **p** | **η²** |
|  |  |  | **Velocity** |  |  |  |
| Emotion | 482029.1900 | 3 | 160676.3967 | 10.8744 | <.001 | 0.426 |
| Residuals | 650129.1498 | 44 | 14775.6625 |  |  |  |
| **Acceleration** | | | | | | |
| Emotion | 8530.0827 | 3 | 2843.3609 | 0.9325 | 0.4331 | 0.06 |
| Residuals | 134166.1535 | 44 | 3049.23084 |  |  |  |
| **Vertical Movement** | | | | | | |
| Emotion | 2361923.7532 | 3 | 787307.9177 | 21.6891 | < .001 | 0.5966 |
| Residuals | 1597188.0900 | 44 | 36299.7293 |  |  |  |
| **Limb Angles** | | | | | | |
| Emotion | 246.1839 | 3 | 82.0613 | 0.6851 | 0.5659 | 0.0446 |
| Residuals | 5270.0116 | 44 | 119.7730 |  |  |  |
| **Limb Contraction** | | | | | | |
| Emotion | 30124.0751 | 3 | 10041.3584 | 1.3517 | 0.27 | 0.0844 |
| Residuals | 326858.0118 | 44 | 7428.5912 |  |  |  |
| **Symmetry** | | | | | | |
| Emotion | 1595.6612 | 3 | 531.8871 | 1.4553 | 0.2397 | 0.0903 |
| Residuals | 16081.0995 | 44 | 365.4795 |  |  |  |
| **Volume Average** | | | | | | |
| Emotion | 0.2079 | 3 | 0.0693 | 5.6647 | < .01 | 0.2786 |
| Residuals | 0.5383 | 44 | 0.0122 |  |  |  |
| **Volume Standard Deviation** | | | | | | |
| Emotion | 0.0317 | 3 | 0.0106 | 5.5800 | <.01 | 0.2756 |
| Residuals | 0.0832 | 44 | 0.0019 |  |  |  |
|  | | | | | | |
| **Interpersonal Features** | | | | | | |
|  |  |  |  |  |  |  |
| **Cases** | **Sum of Squares** | **df** | **Mean Square** | **F** | **p** | **η²** |
| **Distance Correlation Velocity** | | | | | | |
| Emotion | 0.2161 | 3 | 0.07203 | 2.6776 | 0.0586 | 0.1544 |
| Residuals | 1.1837 | 44 | 0.02690 |  |  |  |
| **Distance Correlation Acceleration** | | | | | | |
| Emotion | 0.0134 | 3 | 0.0045 | 1.9844 | 0.1302 | 0.1192 |
| Residuals | 0.0989 | 44 | 0.0022 |  |  |  |
| **Distance Correlation Limb Contraction** | | | | | | |
| Emotion | 0.5849 | 3 | 0.1950 | 4.1803 | <.05 | 0.2218 |
| Residuals | 2.0522 | 44 | 0.0466 |  |  |  |
| **Cases** | **Sum of Squares** | **df** | **Mean Square** | **F** | **p** | **η²** |
| **Distance Correlation Volume** | | | | | | |
| Emotion | 0.5737 | 3 | 0.1912 | 3.4853 | <.05 | 0.1920 |
| Residuals | 2.4143 | 44 | 0.0549 |  |  |  |
| **Interpersonal Distance Average** | | | | | | |
| Emotion | 2061978.8989 | 3 | 687326.2996 | 8.4657 | <.001 | 0.3660 |
| Residuals | 3572320.4774 | 44 | 81189.1018 |  |  |  |
| **Interpersonal Distance Standard Deviation** | | | | | | |
| Emotion | 37563.4382 | 3 | 12521.1461 | 3.0221 | <.05 | 0.1708 |
| Residuals | 182303.4471 | 44 | 4143.2602 |  |  |  |
| **Interpersonal Orientation Average** | | | | | | |
| Emotion | 21261.0869 | 3 | 7087.02897 | 9.0113 | <.001 | 0.3806 |
| Residuals | 34604.2070 | 44 | 786.4593 |  |  |  |
| **Interpersonal Orientation Balance** | | | | | | |
| Emotion | 3.2234 | 3 | 1.0745 | 8.4244 | <.001 | 0.3648 |
| Residuals | 5.6119 | 44 | 0.1275 |  |  |  |
| **Motion Energy balance** | | | | | | |
| Emotion | 0.2719 | 3 | 0.0906 | 6.8339 | <.001 | 0.3178 |
| Residuals | 0.5835 | 44 | 0.0133 |  |  |  |
| **Personal Space** | | | | | | |
| Emotion | 29306.1263 | 3 | 9768.7088 | 7.4924 | <.001 | 0.3381 |
| Residuals | 57367.6000 | 44 | 1303.8082 |  |  |  |
| **Synchronization Velocity** | | | | | | |
| Emotion | 0.1801 | 3 | 0.0600 | 2.9495 | <.05 | 0.1674 |
| Residuals | 0.8954 | 44 | 0.0204 |  |  |  |
| **Synchronization Acceleration** | | | | | | |
| Emotion | 0.0472 | 3 | 0.0157 | 1.9378 | 0.1374 | 0.1167 |
| Residuals | 0.3571 | 44 | 0.0081 |  |  |  |

| **S2 Table. Feature and Behavioural Rating RDM Relatedness**  **Feature and Emotion RDM Relatedness** | | | |
| --- | --- | --- | --- |
| **Intrapersonal Features** | **r** | **SE** | **p** |
| Vertical Movement | 0.1006 | 0.0051 | <0.001 |
| Velocity | 0.0755 | 0.0041 | <0.001 |
| Limb Contraction | 0.0736 | 0.0032 | <0.001 |
| Limb Angles | 0.0686 | 0.0031 | <0.001 |
| Volume Average | 0.0503 | 0.0031 | <0.001 |
| Volume Standard Deviation | 0.0475 | 0.0027 | <0.001 |
| Symmetry | 0.0204 | 0.0017 | <0.001 |
| Acceleration | 0.0038 | 0.0028 | n.s* |
|  | | | |
| **Interpersonal Features** | **r** | **SE** | **p** |
| Personal Space | 0.0685 | 0.0035 | <0.001 |
| IPO Balance | 0.0623 | 0.0039 | <0.001 |
| Motion Energy Balance | 0.0611 | 0.0027 | <0.001 |
| IPD Average | 0.0594 | 0.0036 | <0.001 |
| IPO Average | 0.0562 | 0.0030 | <0.001 |
| DC Limb Contraction | 0.0262 | 0.0024 | <0.001 |
| DC Volume | 0.0168 | 0.0028 | <0.001 |
| Synchronization Acceleration | 0.0146 | 0.0019 | <0.001 |
| IPD Standard Deviation | 0.0140 | 0.0028 | <0.001 |
| Synchronization Velocity | 0.0099 | 0.0017 | <0.001 |
| DC Acceleration | 0.0045 | 0.0023 | <0.01 |
| DC Velocity | 0 | 0.0017 | n.s |
|  |  |  |  |
| **Combination Features** | **r** | **SE** | **p** |
| VM + ME BAL | 0.1109 | 0.0040 | <0.001 |
| VM + IPO | 0.1103 | 0.0041 | <0.001 |
| VM + PS | 0.1035 | 0.0046 | <0.001 |
| VM + IPO BAL | 0.1030 | 0.0044 | <0.001 |
| VM + IPD | 0.1029 | 0.0051 | <0.001 |
| VM + DC LC | 0.0907 | 0.0040 | <0.001 |
|  |  |  |  |
| **Feature and Valence RDM Relatedness** | | | |
| **Intrapersonal Features** | **r** | **SE** | **p** |
| Limb Angles | 0.1155 | 0.0117 | <0.001 |
| Limb Contraction | 0.0823 | 0.0115 | <0.001 |
| Volume Average | 0.0419 | 0.0119 | <0.01 |
| Volume STD | 0.0362 | 0.0081 | <0.001 |
| Vertical Movement | 0.0316 | 0.0098 | <0.01 |
| Velocity | 0.0284 | 0.0066 | <0.001 |
| Symmetry | 0.0260 | 0.0110 | <0.05 |
| Acceleration | 0.0258 | 0.0100 | <0.05 |
|  | | | |
| **Interpersonal Features** | **r** | **SE** | **p** |
| IPO Balance | 0.1806 | 0.0114 | <0.001 |
| Motion Energy Balance | 0.1748 | 0.0107 | <0.001 |
| IPO Average | 0.1107 | 0.0102 | <0.001 |
| IPD Average | 0.0698 | 0.0086 | <0.001 |
| Personal Space | 0.0588 | 0.0068 | <0.001 |
| IPD STD | 0.0485 | 0.0093 | <0.001 |
| Synchronization Velocity | 0.0399 | 0.0100 | <0.001 |
| Synchronization Acceleration | 0.0328 | 0.0102 | <0.05 |
| DC Volume | 0 | 0.0059 | n.s |
| DC Limb Contraction | -0.0052 | 0.0070 | n.s |
| DC Velocity | -0.0070 | 0.0083 | n.s |
| DC Acceleration | -0.0191 | 0.0090 | n.s |
|  |  |  |  |
| **Combination Features** | **r** | **SE** | **p** |
| LA + IPO BAL | 0.2063 | 0.0118 | <0.001 |
| LC + IPO BAL | 0.1960 | 0.0119 | <0.001 |
| LA + ME BAL | 0.1801 | 0.0122 | <0.001 |
| LC + ME BAL | 0.1592 | 0.0117 | <0.001 |
| LA + IPO | 0.1484 | 0.0104 | <0.001 |
| LC + IPO | 0.1368 | 0.0105 | <0.001 |

*n.s = not significant


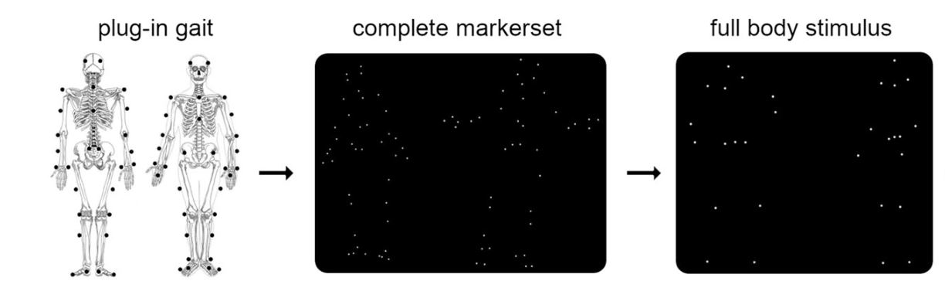
**Stimulus creation**

**S11 Fig. Stimulus creation** Point-Light Displays were created using Vicon Motion Capture System with 41 markers attached to predefined anatomical landmarks. Full-body-stimuli consisted of 15 markers.

**Stimulus creation and Feature Definition.** Features were computed for each stimulus in a stimulus dataset comprising 48 emotional interactions expressing happiness, affection, sadness and anger. Consequently, features were calculated from the x-, y-, and z-coordinates. From the 15 markers displayed, 13 anatomical points were determined (excluding sternum and sacrum), representing anatomical landmarks on the upper body (including the shoulders, elbows, wrists and the forehead) and the lower body (including the hips, knees and ankles). For example, the left hip marker was calculated by averaging the x-, and y-coordinates of the left anterior superior iliac (LASI) & left posterior superior iliac (LPSI) (“Plug-in-Gait”) and height was determined by the z component of the LASI. Left wrist marker A (LWRIA) and left wrist marker B (LWRIB) were averaged to compute the joint center of wrist segment. The equivalent was done for the right hip and wrist. Coordinates of the head marker were assessed by averaging the right forehead (RFHD), left forehead (LFHD), right back of head (RBHD) and left back of head (LBHD) coordinates. This results in the following order of the anatomical points: HEAD, left shoulder (LSHO), left elbow (LELB), left wrist (LWRI), left hip (LHIP), left knee (LKNE), left ankle (LANK), right shoulder (RSHO), right elbow (RELB), right wrist (RWRI), right hip (RHIP), right knee (RKNE), right ankle (RANK) for both actors.

**Intrapersonal Features**Kinematic Features

*Velocity* was calculated for every single marker from the 3-dimensional motion trajectories divided by the according time interval (1/100 Hz = 10 ms). *Acceleration* was then derived from the calculated velocity. *Vertical movement* was computed as the absolute amount of displacement of each marker in the z-axis between adjacent frames. Kinematic features were evaluated for each marker (26) and averaged across time and people resulting in 13 values for each feature.

#### Postural Features

The postural feature *symmetry* was calculated in three steps. Firstly, the difference in height (z-axis) between the left and right side of the bodies was assessed for the corresponding markers e.g. LWRI and RWRI. A line of symmetry was then determined considering the position of the shoulder and hip markers in the x and y coordinates. These were used to find one middle point between the hips and one between the shoulders and furthermore to calculate an artificial point between the centre of those. These calculations divide the body in the middle and enables us to take possible twists and rotations in the proximal torso area into consideration. A vector was created from this centre to the front of the body and the distance of the right and left markers to the resulting line of symmetry was calculated. In addition, the resulting angle between the symmetry point and the corresponding markers on both sides were determined and transformed into the circular segment.


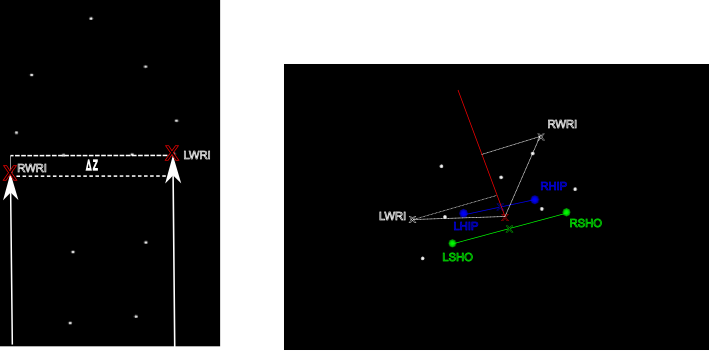


**S12 Fig. Symmetry measures.** Left figure shows symmetry in z-direction (height) of the wrist points. Right figure ilustrates symmetry in x-, and y-direction as well as the corrsponding line of symmetry for one person at one frame.

Finally, the absolute difference of the two sides of the body with regard to the three symmetry measures (height, distance from the symmetry line and circular segment) was determined and averaged across actors resulting in 18 values (6 markers on each side of the body * 3 symmetry measures).

*Limb Angles* include the shoulder, elbow, hip and knee joint angles. For example, the shoulder angle on the left side of the body is calculated as the included angle from the LSHO to the LELB and LHIP. Whereas, the elbow angle is defined between the LSHO, LELB and LWRI marker. Of the two angles on the lower body, the hip angle describes the angle between the vectors of LHIP to LSHO and LKNEE. The knee angle lies between the vectors of RKNE to RHIP and RANK. All described angles were determined on both sides of the body resulting in eight values.

**S13Fig. Limb angles and limb contraction** with corresponding antomical points


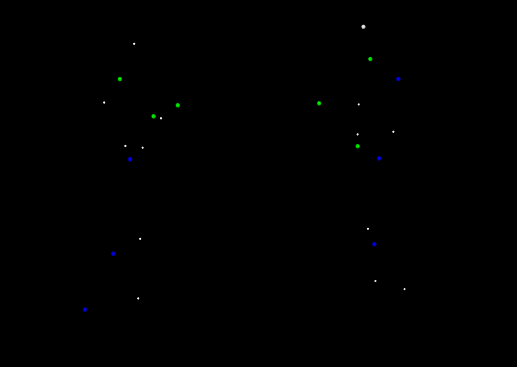

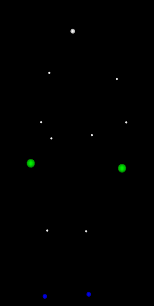


HEAD

RWRI

LWRI

RANK

LANK

RANK

RHIP

RKNE

RHIP

RSHO

LSHO

LELB

LWRI

RWRI

LSHO

LHIP

LKNE

The feature *Limb Contraction* included the distances from the LANK and RANK and the LWRI and RWRI to the HEAD resulting in four values for each stimulus. Volume was computed as the multiplication of the distances between the minimum and maximum anatomical point of a person along the x-, y-, and z-axes for each time stamp. To investigate the dynamic change in *volume*, the standard deviation of the calculated volume was included as well, resulting in one value for *volume* and *volume standard deviation* for each stimulus.

### Interpersonal Features

The interaction-specific character of the stimuli was described through different parameters. Firstly, the interpersonal distance was computed by calculating an artificial center for each person between the LHIP and RHIP and later used to calculate the Euclidean distance between both center points at each frame. The result was then averaged over time while the variability between the two actors regarding their distance was preserved in the standard deviation.


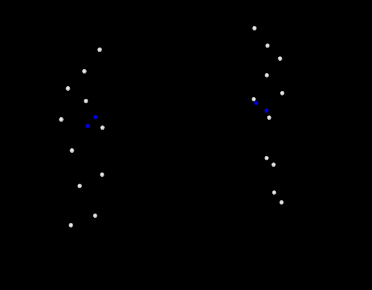


IPD

**S14 Fig. Interpersonal distance** measure for one stimuli at one frame

The parameter *distance correlation* was presented to examine the extent to which distance influences various individual kinematic and postural features. For this purpose, the distance over time was correlated (Pearson) with the velocity and acceleration profile as well as with the volume and limb contraction. Because the noisy velocity and acceleration profiles would influence the correlation, a moving average filter (window size = 10 dp / 100 ms) was applied for kinematic features. In the next step, the absolute values of the correlations were calculated and averaged across people. Due to violations of normal distribution in the correlation values Fisher Z-Transformation was applied before averaging across people and transformed back afterwards. Thus, resulting in 13 correlation values for the kinematic *distance correlation* values and respectively four limb contraction and one volume postural *distance correlation* values.


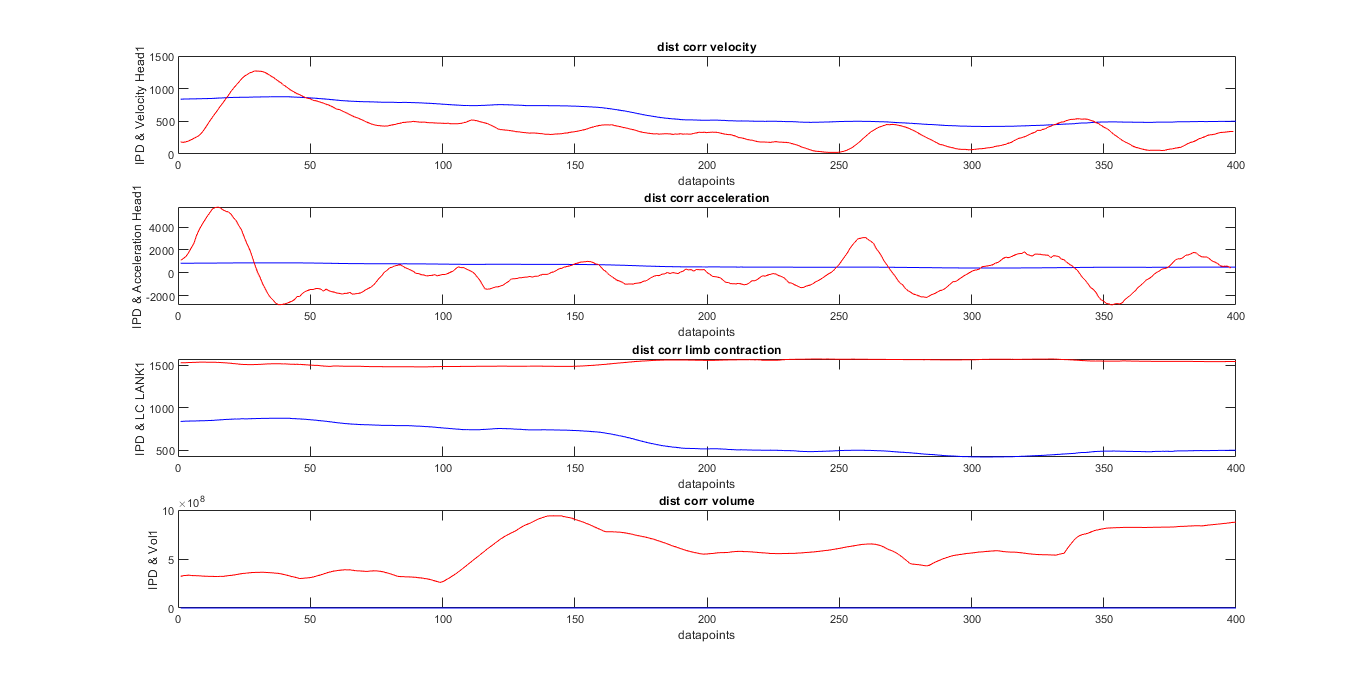


**S15 Fig. Distance correlation**. IPD profile and corresponding kinematic (velocity & acceleration of one anatomical point) profile as well as postural (limb contraction and volume) profile over time

*Personal space* was inspired by the work of Edward T. Hall as he noticed that “people like to keep certain distances between themselves and other people”. Even though this interpersonal distance has already been described via the IPD parameter, Hall referred to a “bubble that surrounds the organism” (Overhill, 2014; Sorokowska et al., 2017) and distinguished between four dimensions. Hall’s research indicates a spatial hierarchy starting from intimate to personal, social and public. In a first approximation, he also defined spatial boundaries between the zones but also noticed that his research was limited. Therefore, we have chosen a different approach to calculating the personal zone, which does not work with "fixed boundaries" but considers the physicality of the interacting subjects. As Overhill (2016) highlights, getting close to each other, within arm’s length, is risky but also important in a cultural symbolic meaning expressed by a handshake or the social kiss. Therefore, we used the arm length to define a personal area for each person. The arm length was calculated from the distance of LSHO to LELB and LELB to LWRI and was averaged for both sides of the body. We also averaged arm lengths of the interacting subjects under the assumption that both are crucial for action and reaction. Moreover, we assumed that the proximity to the core (defined by the hips and shoulders) of the opposite person is particularly important. The calculation of time spent in the other persons space was done in two steps. First, we defined a polygon in the front and back of the hips and shoulders, spatially limited by the computed arm length, and checked if each marker from the other person lies in this predefined space (see Fig. SM 17 upper figure). In the second step, we calculated the Euclidean distance of every marker of a person in relation to the core markers and tested if the distance is smaller or equal to the predefined arm length (see Fig. SM 17 lower figure). The percentage of time in each other's space over the total stimulus duration was then determined for both persons and averaged.


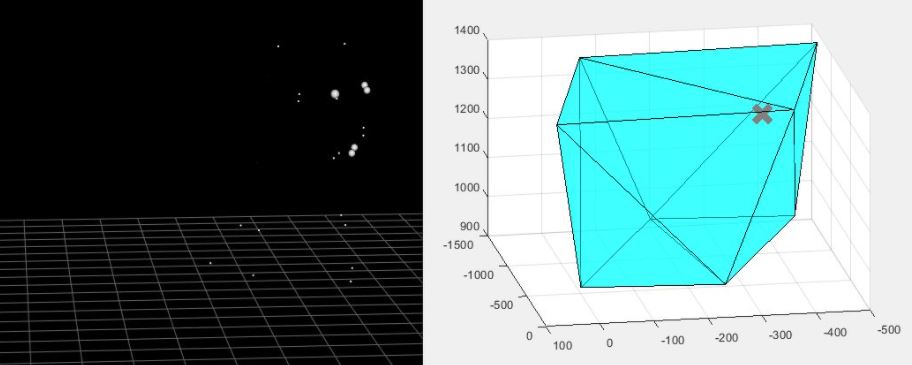


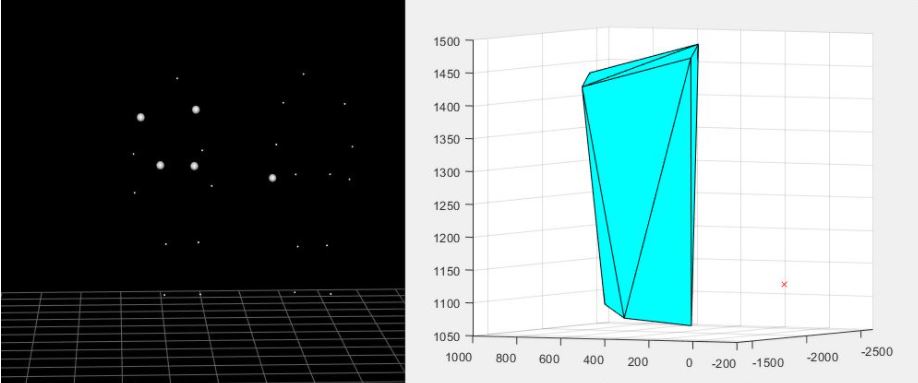


**S16 Fig. Personal space**. The upper picture shows the penetration of one person into the personal space (polygon) of the others. The lower picture illustrates the opposite. Euclidean distance measure is depicted in the upper left figure for calculation of RWRI and corresponding core markers.

Further, the *synchronization* of the interacting persons was determined. In everyday life, the importance of synchronization of non-verbal communication occurs in many areas like the interaction between a mother and infant, physician and patient, teacher and student or psychological counsellor and client (Kohne et al., 2016; Thepsoonthorn et al., 2016; Yokozuka et al., 2018). Yokozuka and colleagues as well as Thepsoonthorn and colleagues measured the acceleration of speakers and listeners head motion synchronization in a unidirectional face-to-face communication task with a duration of about five minutes. Koehne and colleagues investigated unilateral synchrony in leader-follower tapping communication tasks in individuals with and without autism spectrum disorder. In contrast to the literature described, our presented interactions were not divided into an active and responding role and took only 4 seconds. Thus, we did not transform the parameters to the frequency spectrum but rather searched for synchronicity within the velocity and acceleration profiles time domain. Due to the lack of role assignment, we checked the correlation (Pearson) of the velocity and acceleration profiles of both actors over a moving time window of 20 ms (2 data points) for the duration of one second. Hence, the noisy velocity and acceleration profiles would also

**S17 Fig. Moving time lag window.** synchronization for both HEAD markers and shifted velocity profiles of actor one (middle) and actor two (lower). The shown time lag amounts 10 datapoints.

*
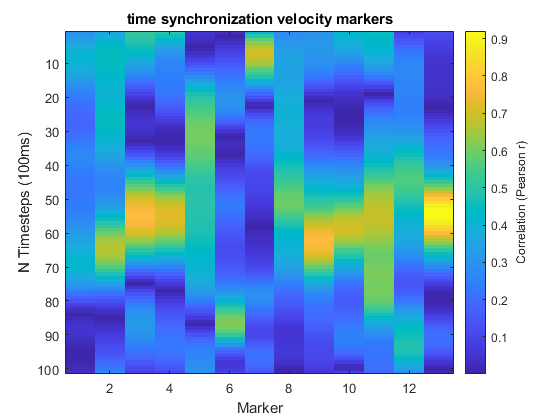
*influence the correlation of this feature. A moving average filter (window size = 10 dp / 100 ms) was applied as in the distance correlation feature. The profiles of both actors were temporally shifted and then correlated as depicted in Fig. SM 18 for the HEAD markers velocity profile for each corresponding marker resulting in a number of timesteps * number of markers correlation matrix as shown in Figure SM 19. Absolute correlations were computed under the assumption that negative correlations are also an indicator for interaction. In the last step, the maximum correlation was determined for each corresponding marker resulting in 13 correlation values beginning by the HEAD marker and ending with the RANK.

**S18 Fig. Synchronization profile.** Incidence of atomical keypoint velocity synchronization for one stimuli

Synchronization profiles illustrates the variability of the correlation depending on predefined anatomical points and timesteps. Due to violations of normal distribution in the correlation values Fisher Z Transformation was applied. To use this variability and explore the emotional information content of different body segments, synchronicity was specified and, following Bachmann and colleagues (2020), divided into two areas. On one hand, ‘arm’ segments are defined via the shoulders, elbows and wrist markers of the persons and examined regarding their synchronicity. On the other hand, the same procedure is done for ‘trunk’ segments consisting of the head, hip, knee and ankle joint markers (*Sync Vel Arm & Sync Vel Trunk; Sync Acc Arm & Sync Acc Trunk*).

Research has proven that body orientation is significantly affected by a communicator’s attitude towards the interaction partner (Kleinsmith and Bianchi-Berthouze, 2013). This finding was used in the *interpersonal orientation* parameter. The orientation of both actors was calculated by the positions of the shoulder markers. In the first step, artificial center points (ACP) between the LSHO and RSHO from both actors were computed and the maximal Euclidean distances between each shoulder marker were calculated. The resulting maximal distance (1.1 * max. shoulder distance, to ensure sufficient length) was used to calculate an orthogonal vector, pointing forward from each shoulder marker. In the resulting corridor, the minimal distance between the ACP of person one and the orientation vectors of person two was then determined and compared with the maximal distance between the corresponding shoulder markers of person one. The resulting timeseries was later averaged across time and people. If one person was oriented towards the other for a longer period, this imbalance was preserved via the IPO balance. Balance is calculated by 1 - the absolute value of the difference between the orientation times divided by the sum of the orientation times. Hence, minor balance values indicate small balance and vice versa.


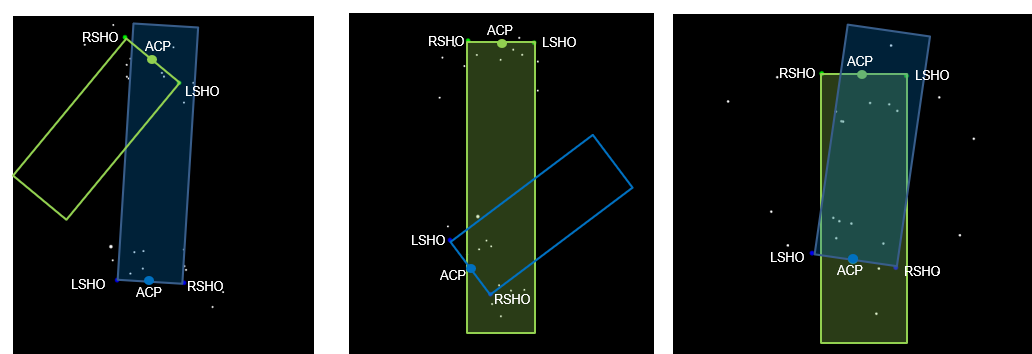


**S19 Fig. Interpersonal orientation**. Person 1 (blue), Person 2 (green) with corresponding orientation corridors (length ratios have been adjusted for illustration). Left figure depicts orientation from person 1 to person 2 and middle figure vice versa. Right figure ilustrates Face-To-Face orientation.

*Motion energy* was used by Thurman and Lu (2014) to discriminate between animate and inanimate interactions. Also, Kleinsmith and Bianchi-Berthouze (2013) highlighted that for example sadness and shame are characterized by slow, low energy movements. Therefore, we calculated the motion energy displayed by each actor as the sum of the averaged inter-frame Euclidean displacement of each marker (Thurman and Lu, 2014). To emphasize the interaction-specific part, we determined the motion-energy-balance displayed by both actors in the same way we calculated IPO balance. Analogously, high values indicate a strong balance between the displayed motion energy of both actors.
